# Engineered probiotic-derived indole-3-propionic acid inhibits ubiquitination via AHR signaling to treat postmenopausal osteoporosis

**DOI:** 10.1080/19490976.2025.2612620

**Published:** 2026-01-08

**Authors:** Xueli Qiu, Lijun Wu, Fengxian Jiang, Huajian Shan, Lei Sheng, Bo Tian, Heng Wang, Hao Cui, Lide Tao, Chenyang Wu, Yuqian Yao, Chao Wang, Xiaozhong Zhou, Yingzi Zhang, Jinyu Bai

**Affiliations:** a Department of Orthopedics, The Second Affiliated Hospital of Soochow University, Suzhou, China; b Department of Plastic and Aesthetic Surgery, The Second Affiliated Hospital of Soochow University, Suzhou, China; c Institute of Functional Nano & Soft Materials (FUNSOM), Soochow University, Suzhou, China

**Keywords:** Postmenopausal osteoporosis, gut microbiota, indole-3-propionic acid, gut-bone axis, engineered probiotic

## Abstract

Postmenopausal osteoporosis (PMOP) has a high incidence in middle-aged and elderly women, leading to an increased risk of fractures and elevated rates of disability and mortality. In this work, we identified the reduction of indole-3-propionic acid (IPA) as a potential key factor contributing to the decline in bone mass observed in postmenopausal women. Mechanistically, IPA activates AhR, leading to the stabilization of key proteins in Wnt and NF-κB pathways that regulate bone formation and resorption. We evaluated efficacy *in vivo* using eight-week-old female C57BL/6 mice subjected to bilateral ovariectomy (OVX), with treatments initiated one week postsurgery, and performed complementary *in vitro* assays. Intraperitoneal IPA (20 mg/kg, 3× per week for 8 weeks) increased the trabecular bone mineral density (Tb.BMD) by ~68% versus OVX controls, whereas engineered *Clostridium sporogenes* that enhances IPA biosynthesis led to an even greater increase of ~118%. Together, these findings highlight the gut–bone axis as a central framework linking microbiota-derived IPA to skeletal remodeling and provide preclinical proof-of-concept for an engineered *C. sporogenes*–IPA strategy with therapeutic potential in PMOP.

## GRAPHICAL ABSTRACT

**Figure:**
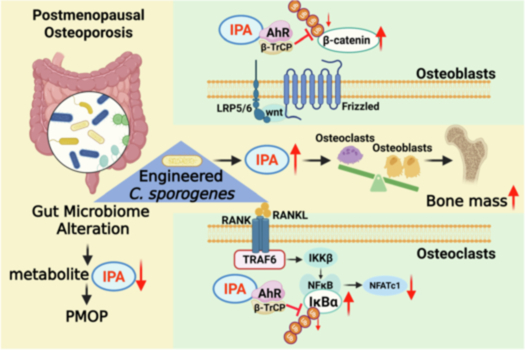

The link between gut microbiota Tryptophan metabolite IPA and PMOP, and its mechanism in regulating bone metabolism.

## Introduction

Postmenopausal osteoporosis (PMOP) is a common metabolic bone disorder characterized by reduced bone mineral density (BMD) and deterioration of trabecular architecture, caused by an imbalance between osteoclast-mediated bone resorption and osteoblast-driven bone formation.^1^ More than 200 million people worldwide suffer from osteoporosis, and postmenopausal women face the highest lifetime fracture risk.^2^ Up to 40% will experience an osteoporotic fracture after age 50, and hip fractures in particular lead to profound disability and increased mortality.^3^ Although estrogen deficiency is the primary driver of PMOP, the wide interindividual variability in bone loss points to additional biological processes shape skeletal vulnerability after menopause.^4^

Growing evidence suggests that the gut–bone axis contributes to the regulation of skeletal homeostasis in PMOP.^5^ Gut dysbiosis, intestinal inflammation, and impaired epithelial barrier function have been associated with altered osteoimmune signaling and disrupted bone remodeling.^6^^,^^7^ The gut microbiota interacts with distant organs partly through bioactive metabolites that can enter the circulation.^8^ Several well-characterized metabolites—including short-chain fatty acids (SCFAs), trimethylamine N-oxide (TMAO), and bile acids—have been closely linked to the development and progression of osteoporosis.^9-11^ More recently, amino-acid-derived microbial metabolites, particularly those from tryptophan metabolism, have gained attention for their broad roles in regulating mucosal immunity and metabolic homeostasis.^12^^,^^13^ However, how tryptophan metabolism is altered in PMOP—and whether such alterations contribute to bone loss—remains incompletely understood.

Indole-3-propionic acid (IPA) is a microbiota-exclusive tryptophan metabolite produced predominantly by *Clostridium sporogenes* and is recognized for its anti-inflammatory, immunomodulatory, and barrier-protective functions.^14-16^ Lower circulating IPA levels have been associated with systemic metabolic dysfunction, including increased risk of type 2 diabetes.^17^ IPA also alleviates rheumatoid arthritis and suppresses chondrocyte inflammation while ameliorating osteoarthritis progression,^18^^,^^19^ suggesting its broader relevance in skeletal disorders. Moreover, microbiome analyses by Rettedal et al. and Wang et al. revealed that the abundance of *Clostridia* species is positively associated with PMOP.^20,21^ Given that *C. sporogenes* is the principal microbial source of IPA, reduced *Clostridia* abundance in PMOP may imply decreased IPA production, linking gut dysbiosis to skeletal deterioration and suggesting a potentially important role for IPA in PMOP.

The aryl hydrocarbon receptor (AhR) is a ligand-activated cytosolic receptor broadly expressed across mammalian tissues and has been implicated in diverse physiological processes, including the regulation of skeletal homeostasis.^22^ Several tryptophan-derived indole metabolites serve as endogenous AhR ligands and can modulate cellular functions by affecting downstream signaling proteins.^23^^,^^24^ Emerging studies further indicate that AhR signaling can intersect with major intracellular pathways, including Wnt and NF-κB, both of which are central regulators of osteoblast and osteoclast function.^25^ Beyond its canonical transcriptional roles, AhR also contributes to the regulation of protein stability through ubiquitination-related mechanisms. For example, β-indole-3-acetic acid (IAA) reduces Foxp3 ubiquitination via an AhR–TAZ–Tip60 axis, thereby stabilizing Foxp3 protein levels.^26^ These observations led us to hypothesize that IPA may likewise activate AhR to suppress the ubiquitination-dependent degradation of key proteins involved in bone remodeling.

Clinically, PMOP is managed with antiresorptive and anabolic agents, yet limitations related to long-term safety, cost, and patient adherence highlight the need for alternative, noninvasive therapeutic approaches.^27^ Recent systematic reviews and meta-analyses—particularly those conducted in human populations—have provided milestone evaluations of probiotic interventions in postmenopausal osteoporosis, collectively indicating promising benefits on calcium handling, bone turnover markers, and bone mineral density.^28-30^ With rapid advances in synthetic biology and gene engineering, the development of engineered probiotics has emerged as a frontier strategy for treating diverse diseases.^31^ Motivated by this evidence, we considered whether engineering a native gut commensal could be leveraged to enhance the production of a bone-regulating microbial metabolite. We therefore focused on *C. sporogenes*, a natural producer of IPA with demonstrated probiotic potential, including benefits for muscle development and cognitive function in mice,^32^^,^^33^ as a candidate chassis for such an approach. Nevertheless, the clinical translation of engineered probiotics remains constrained by challenges such as strain stability, metabolic control, biosafety, and interhost variability in terms of colonization and metabolite output.

In this study, we characterized alterations in tryptophan metabolism in PMOP and identified IPA as a key microbiota-derived metabolite that supports bone homeostasis by coordinating the coupling between bone formation and resorption. Building on this principal discovery, we engineered a *C. sporogenes* strain to enhance intestinal IPA biosynthesis and showed that it alleviates bone loss in OVX mice, pointing to a noninvasive microbial strategy with therapeutic promise for PMOP.

## Results

### PMOP patients exhibit tryptophan metabolism disorders and reduced IPA levels

**Figure 1.**
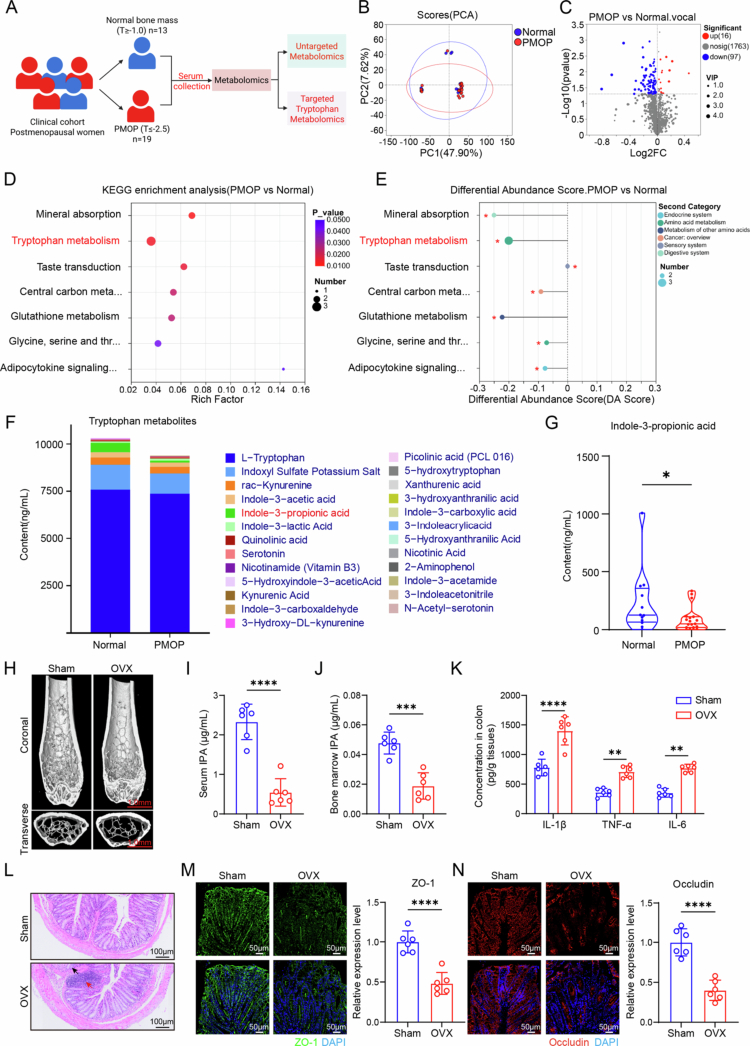
PMOP patients exhibit tryptophan metabolism disorders and reduced IPA levels; similarly, OVX mice show decreased IPA levels and impaired intestinal barrier function. (A) Schematic diagram of collecting serum from postmenopausal women for metabolomics sequencing. (B) Principal component analysis (PCA) shows metabolic differences between 19 PMOP patients (PMOP, red) and 13 postmenopausal women with normal bone mass (normal, blue). (C) Volcano plot showing differential metabolite expression between the PMOP and normal groups. A log2 (fold change) threshold of 1 and a *p*-value of 0.05 identified 97 downregulated and 16 upregulated metabolites. (D) KEGG pathway enrichment analysis of differential metabolites. (E) Differential abundance score plots of KEGG pathways. Negative values indicate downregulation, and positive values indicate upregulation. (F) Serum tryptophan-targeted metabolomics analysis showing quantitative analysis of tryptophan metabolites between PMOP patients and the normal group. (G) The serum IPA levels were measured using LC–MS/MS (normal: *n* = 12; PMOP: *n* = 19; the maximum value in the normal group was removed because the difference was too large). (H) Representative 3D reconstructed images of the distal femur of the mice in each group (scale bar = 1 mm, *n* = 6). (I and J) Serum and bone marrow IPA levels in each group (*n* = 6). (K) Levels of inflammatory cytokines in intestinal tissue (*n* = 6). (L) Representative hematoxylin and eosin (H&E)-stained sections of intestinal tissue: red arrows indicate inflammatory cell infiltration; black arrows show crypt structure disorganization and goblet cell loss (scale bar = 100 µm, *n* = 6). (M and N) Representative images and quantitative analysis of tight junction proteins ZO-1 and Occludin expression in colon tissues (scale bar = 50 µm, *n* = 6). The data are presented as mean ± SD. Statistical significance was obtained by Student's *t* test (two-tailed) (I, J, K, M, and N) and Mann–Whitney *U* test (G). Significance: *p < 0.05; **p < 0.01; and ***p < 0.001.

To investigate if gut microbiota metabolites influence bone metabolism, we collected serum samples from PMOP patients (*n* = 19) and postmenopausal women with normal bone mass (*n* = 13) for untargeted metabolomics analysis (Figure 1A). Principal component analysis (PCA) revealed 55.52% of the total variation between the two sample groups (Figure 1B). Partial least squares-discriminant analysis (PLS-DA) and orthogonal partial least squares-discriminant analysis (OPLS-DA) (Figure S1A and B) further separated the relevant and irrelevant variation between the two groups. Furthermore, a total of 113 differentially expressed metabolites (DEMs) were identified, 97 of which were downregulated by more than two-fold, and 16 DEMs were upregulated (Figure 1C). Noteworthy, KEGG enrichment analysis showed that the tryptophan metabolism pathway was significantly perturbed, with several pathway intermediates markedly downregulated in PMOP patients (e.g., N-formylanthranilic acid, 2-(1H-indol-3-yl)acetaldehyde, and 5-(2′-carboxyethyl)-4,6-dihydroxypicolinate) (Figures 1D and S2A). KEGG pathway differential abundance score plots showed a negative differential abundance value, indicating tryptophan metabolism dysfunction in PMOP patients (Figure 1E). Further targeted tryptophan metabolomic analysis revealed that among 25 tryptophan metabolites, only the IPA levels showed a significant decrease in PMOP patients compared to postmenopausal women with normal bone mass (*p* < 0.05) (Figures 1F,G and S3A). Interestingly, although untargeted metabolomics identified three downregulated metabolites in the tryptophan pathway, targeted validation confirmed a significant alteration only in IPA, likely due to differences in metabolite coverage between the two analytical platforms. This finding suggests that IPA may play a pivotal role in the development and progression of postmenopausal osteoporosis. Taken together, these results show that PMOP patients exhibit tryptophan metabolic disorders and reduced IPA levels.

### OVX mice show decreased IPA levels and impaired intestinal barrier function

To verify whether ovariectomy also led to impaired tryptophan metabolism and decreased IPA levels in animal models, we established an OVX mouse model to simulate postmenopausal conditions. μCT analysis revealed a significant reduction in bone mineral density (BMD), bone volume/total volume (BV/TV), bone surface area to total volume (BS/TV), trabecular thickness (Tb.Th), and trabecular number (Tb.N) in the OVX group compared to the sham group (Figures 1H and S4A–E). In contrast, the trabecular spacing (Tb.Sp) increased (Figure S4F). Then, we assessed IPA concentrations in the serum and bone marrow of the Sham and OVX groups. The results showed a significantly reduced IPA levels in both the serum and bone marrow of OVX mice compared with the sham group (Figure 1I,J), suggesting that IPA may be associated with osteoporosis caused by estrogen deficiency. Consistently, previous targeted metabolomics in OVX mice also revealed tryptophan metabolic disorder, among which IPA showed a significant decrease,^34^ further supporting the reproducibility of our human findings in the OVX model.

Research has reported that estrogen deficiency may induce intestinal inflammation and mucosal damage.^35^ To clarify the impact of estrogen deficiency on the intestinal barrier and function, we analyzed the levels of inflammatory cytokines in colon tissues. Our results revealed a significant upregulation of IL-1β, TNF-α, and IL-6 in OVX mice compared to the Sham group, suggesting that estrogen deficiency induces intestinal inflammation (Figure 1K). Additionally, histological analysis using HE staining showed typical signs of inflammation in the colon of OVX mice, including enterocyte damage and infiltration of inflammatory cells (Figure 1L). Immunofluorescence staining further demonstrated a marked reduction in the expression of the tight junction proteins ZO-1 and Occludin in OVX mice (Figure 1M,​​​​​​​N), indicating a disruption in intestinal barrier integrity. The disruption of intestinal barrier function may elicit chronic inflammation and immune activation that contribute to the development of multiple diseases, including osteoporosis.^35^^,^^36^ Moreover, intestinal barrier function is closely associated with gut microbial metabolism; studies have shown that microbiota-derived molecules such as IPA can influence epithelial junctional architecture and are involved in modulating barrier-associated signaling pathways.^16^ Taken together, these findings suggests that decreased IPA levels and compromised intestinal barrier integrity may contribute to the pathogenesis of postmenopausal osteoporosis.

### IPA supplementation protect mice against OVX-induced bone loss

**Figure 2.**
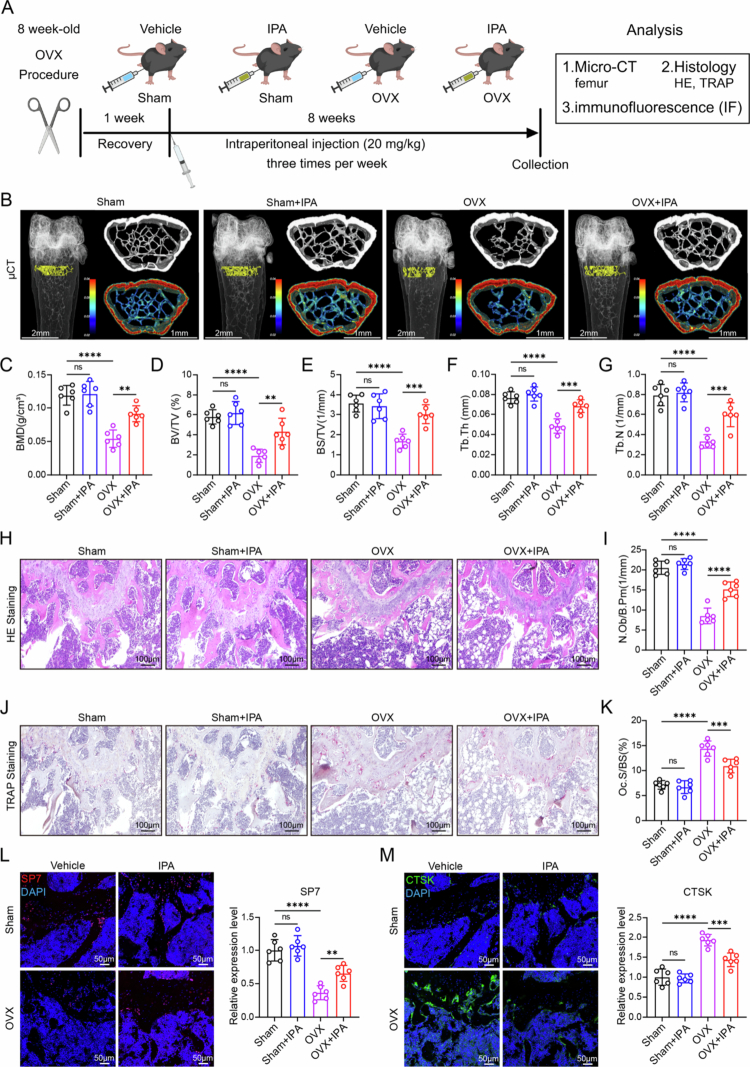
IPA supplementation protect mice against OVX-induced bone loss. (A) Study design of *in vivo* animal experiments. (B) Microcomputed tomography (µCT) 3D reconstruction of the mouse femur (scale bar = 1 mm. *n* = 6). (C–G) Quantitative analysis of bone structural parameters in the distal femur, including bone mineral density (BMD) (C), bone volume/tissue volume (BV/TV) (D), bone surface area/tissue volume (BS/TV) (E), trabecular thickness (Tb.Th) (F), and trabecular number (Tb.N) (G) (*n* = 6). (H and I) H&E staining of distal femur slices in each group, with corresponding quantitative analysis (scale bar = 100 µm, *n* = 6). (J and K) TRAP staining of distal femur slices in each group, with quantitative analysis (scale bar = 100 µm, *n* = 6). (L) Immunofluorescence of SP7 in bone tissue, along with corresponding quantitative analysis (scale bar = 50 µm, *n* = 6). (M) Immunofluorescence of CTSK in bone tissue, with quantitative analysis (scale bar = 50 µm, *n* = 6). The data are presented as mean ± SD. Statistical significance was obtained by one-way ANOVA using the Tukey post-test. Significance: *p < 0.05; **p < 0.01; ***p < 0.001; ****p < 0.0001; and NS, nonsignificance.

To evaluate the effects of IPA on bone mass in mice, mice were divided into four groups: Sham, Sham + IPA, OVX, and OVX + IPA (Figure 2A). IPA (20 mg/kg) or PBS was intraperitoneally injected 3× a week for 8 weeks, followed by micro-CT analysis (Figure 2B). Consistent with expectations, the Sham mice exhibited intact and dense trabecular structures, while the OVX mice displayed significant bone mass loss and trabecular architecture degradation, confirming successful OVX modeling establishment (Figure 2B). There was no significant difference in the bone mass of the mice between the Sham + IPA group and the sham group, suggesting that IPA does not notably affect bone homeostasis under normal physiological conditions. Importantly, compared with the OVX group, the OVX + IPA group showed significant increases in BMD, BV/TV, BS/TV, Tb.Th, and Tb.N (Figure 2C–G), indicating that IPA intervention alleviated bone loss in OVX mice.

These findings were corroborated by histological analyses of femoral sections. H&E and TRAP staining revealed a significant reduction in osteoblast number per bone perimeter (N.Ob/B.Pm) and an increased osteoclast surface-to-bone surface ratio (Oc.S/BS) in OVX mice (Figure 2H–K). In contrast, OVX + IPA-treated mice increased bone trabecular structure and decreased the number of osteoclasts, reducing the severity of bone loss (Figure 2H–K). Moreover, immunofluorescence analysis revealed that the osteoblast-related protein SP7 was significantly reduced in OVX mice but markedly increased in the OVX + IPA group. In contrast, the osteoclast-related protein CTSK was significantly elevated in OVX mice, while it was notably decreased in the OVX + IPA group (Figure 2L,N). These results confirm that IPA supplementation significantly mitigated bone mass loss in OVX mice.

To further validate these findings, we isolated primary bone marrow mesenchymal stem cells (BMSCs) and primary monocytes (BMMs) from the aforementioned mice for subsequent *in vitro* analyses. Alkaline phosphatase (ALP) activity and alizarin red staining (ARS), used to assess osteogenic differentiation and mineralization, respectively, demonstrated that OVX + IPA mice exhibited enhanced osteogenic potential compared to OVX mice (Figure S5A,B). Additionally, the osteoclast differentiation potential of BMMs was evaluated through TRAP and F-actin staining, which revealed increased osteoclast differentiation in OVX mice, while a reduced osteoclast differentiation capacity was observed in the OVX + IPA group (Figure S5C, D). Western blot analysis further revealed that the levels of osteogenesis-related proteins (RUNX2 and SP7) and key components of the osteogenesis-related signaling pathway (β-catenin) were significantly decreased in OVX mice but markedly increased in the OVX + IPA group (Figure S5E). In contrast, proteins associated with osteoclast differentiation, such as CTSK, TRAF6, and NFATc1, were elevated in OVX mice but significantly reduced in the OVX + IPA group (Figure S5F). Collectively, these results demonstrate that IPA supplementation alleviates osteoporosis by modulating bone metabolism and restoring the balance between osteogenesis and osteoclastogenesis. Additionally, we performed a thorough analysis of the major organs in IPA-treated mice, confirming that IPA administration is safe and well tolerated, with negligible toxicity and no significant adverse effects (Figure S6A).

### IPA promotes osteoblastic differentiation and inhibits osteoclastic differentiation *in vitro*

Osteoblasts and osteoclasts work together to maintain proper bone remodeling. To examine the effect of IPA on the differentiation and function of both osteoblasts and osteoclasts *in vitro*, we first performed a CCK-8 assay to determine that IPA concentrations up to 20 μM did not affect the proliferation of BMSCs, MC3T3-E1, BMMs, or RAW cells. This finding ruled out the possibility that IPA promoted osteoblast differentiation by enhancing osteoblast proliferation or inhibited osteoclast differentiation by reducing osteoclast growth (Figure S7A–D).

**Figure 3.**
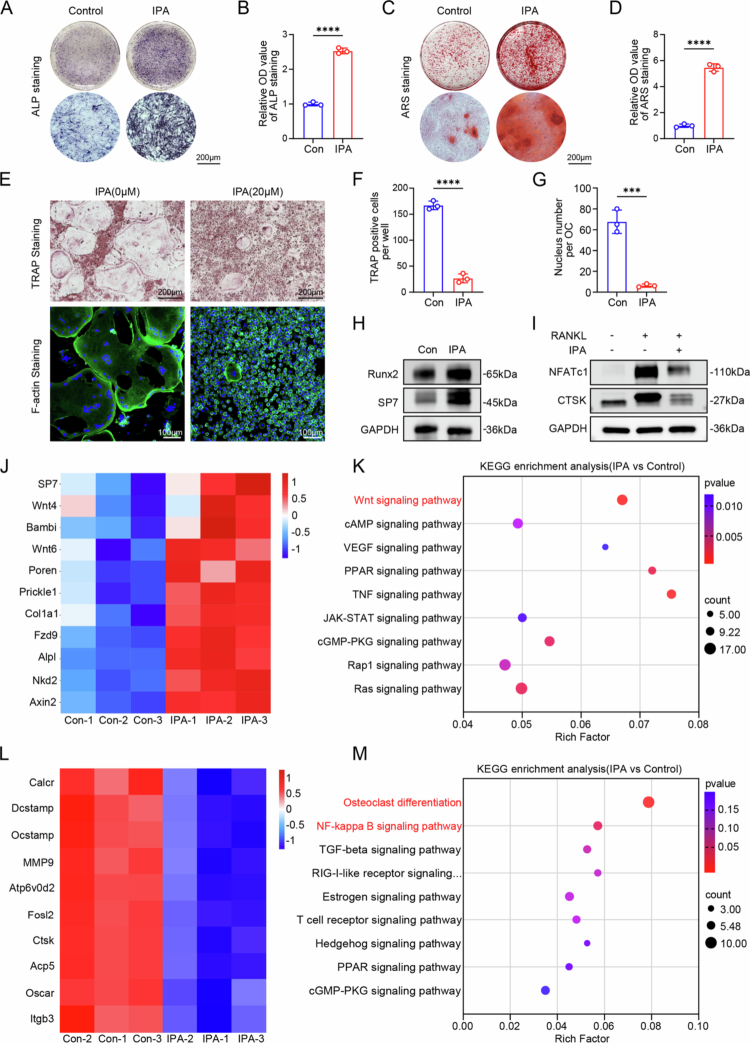
IPA promotes osteoblast differentiation and inhibits osteoclast differentiation *in vitro*. (A) ALP staining of BMSCs treated with IPA (20 μM) (scale bar = 200 µm, *n* = 3). (B) Quantitative analysis of ALP staining intensity, expressed by relative OD value (*n* = 3). (C) ARS staining of BMSCs treated with IPA (20 μM) (scale bar = 200 µm, *n* = 3). (D) Quantitative analysis of ARS staining intensity, expressed by relative OD value (*n* = 3). (E) TRAP staining and F-actin staining of primary monocytes treated with IPA (20 μM) (scale bar = 200 µm or 100 µm, *n* = 3). (F) Quantification of TRAP-positive cells per well (*n* = 3). (G) Quantification of nucleus number per osteoclast (*n* = 3). (H) Representative western blot images showing the effect of IPA on RUNX2 and SP7 protein expression during osteoblast differentiation. (I) Representative western blot images showing the effect of IPA on NFATc1 and CTSK protein expression during osteoclast differentiation. (J) Heatmap showing upregulated genes in the osteogenesis-associated signaling pathway. (K) KEGG enrichment analysis revealed that IPA treatment significantly altered multiple signaling pathways. (L) Heatmap illustrating downregulated genes involved in the osteoclast differentiation signaling pathway. (M) KEGG enrichment analysis showing that IPA treatment significantly altered multiple signaling pathways. The data are presented as mean ± SD. Statistical significance was obtained by Student's *t* test (two-tailed). Significance: ***p < 0.001 and ****p < 0.0001.

Next, we induced osteoblast differentiation in BMSCs. ALP and alizarin red staining revealed that IPA promoted osteoblast differentiation and mineralization in a concentration-dependent manner, with the most pronounced effect observed at 20 μM (Figures 3A–D and S8A,B). For osteoclastic differentiation, primary BMMs were stimulated with RANKL and M-CSF. IPA at concentrations of 0, 5, 10, and 20 μM was added to the culture medium. TRAP and F-actin staining showed that, in the absence of IPA, numerous osteoclasts were formed. However, IPA significantly inhibited osteoclast formation in a dose-dependent manner, with the fewest osteoclasts observed at 20 μM IPA (Figures 3E–G and S8C,D). Subsequent experiments were conducted using IPA at a concentration of 20 μM.

At the molecular level, the effects of IPA on osteogenesis and osteoclastogenesis were confirmed by qPCR analysis of key gene expression. IPA treatment upregulated the expression of early osteoblasts differentiation markers, such as runt-related transcription factor 2 (*Runx2*), collagen type I alpha 1 chain (*Col1a1*) and alkaline phosphatase (*Alpl*). Furthermore, the expression levels of late-stage markers of osteoblast differentiation, such as osteocalcin (*Ocn*), were also notably increased, suggesting that IPA promotes early and late stages of osteoblast differentiation (Figure S9A). In contrast, IPA treatment significantly reduced mRNA expression of osteoclast-specific genes. Including cathepsin K (*Ctsk*), dendritic cell-specific transmembrane protein (*DC-STAMP*), nuclear factor of activated T cells, cytoplasmic 1 (*NFATc1*) and matrix metalloprotein-9 (*MMP-9*) (Figure S9B). Western blot analysis further corroborated that the protein levels of RUNX2 and SP7 were significantly increased in the IPA treatment group compared to the control group during osteoblast differentiation (Figures 3H and S9C). Conversely, the protein expression levels of NFATc1 and CTSK were inhibited in the IPA treatment group (Figures 3I and S9D). These results suggest that IPA interferes with the osteoclast differentiation process by inhibiting their fusion and reducing their activity. These results suggest that IPA has dual functions of promoting osteoblastic differentiation and inhibiting osteoclastic differentiation.

To further investigate the mechanisms underlying the effects of IPA on osteoblast differentiation and osteoclast differentiation, RNA sequencing was performed to examine the transcriptomic changes in osteoblast precursor cells and primary BMMs treated with IPA (20 μM). IPA treatment of osteoblast precursor cells induced significant alterations in 1061 genes expression, including 584 upregulated genes and 477 downregulated genes (Figure S10A). The heatmap analysis revealed that IPA promotes osteogenesis-related genes expression (e.g., *Alpl*, *SP7*, and *Col1a1*) as well as genes associated with the Wnt/β-catenin signaling pathway (e.g., *Wnt4*, *Wnt6*, *Fzd9*, and *Axin2*) (Figure 3J). The KEGG pathway enrichment analysis revealed that IPA treatment significantly enriched the Wnt/β-catenin signaling pathway (Figure 3K). Similarly, IPA treatment of primary BMMs resulted in significant alterations in the expression of 459 genes, including 98 upregulated and 361 downregulated genes (Figure S10B). The heatmap indicated that the expression of key osteoclast-related genes *Ctsk, Oscar, MMP-9, Acp5* and *DC-STAMP* were significantly reduced in the IPA treatment group (Figure 3L). KEGG pathway enrichment analysis further demonstrated that IPA treatment led to significant differential expression in several signaling pathways, including those related to osteoclast differentiation (Figure 3M). In conclusion, IPA may appear to exert a dual regulatory role in bone metabolism by modulating Wnt/β-catenin and NF-κB signaling pathways, which can promote bone formation and inhibit bone resorption.

### IPA regulates bone metabolism by activating AhR

**Figure 4.**
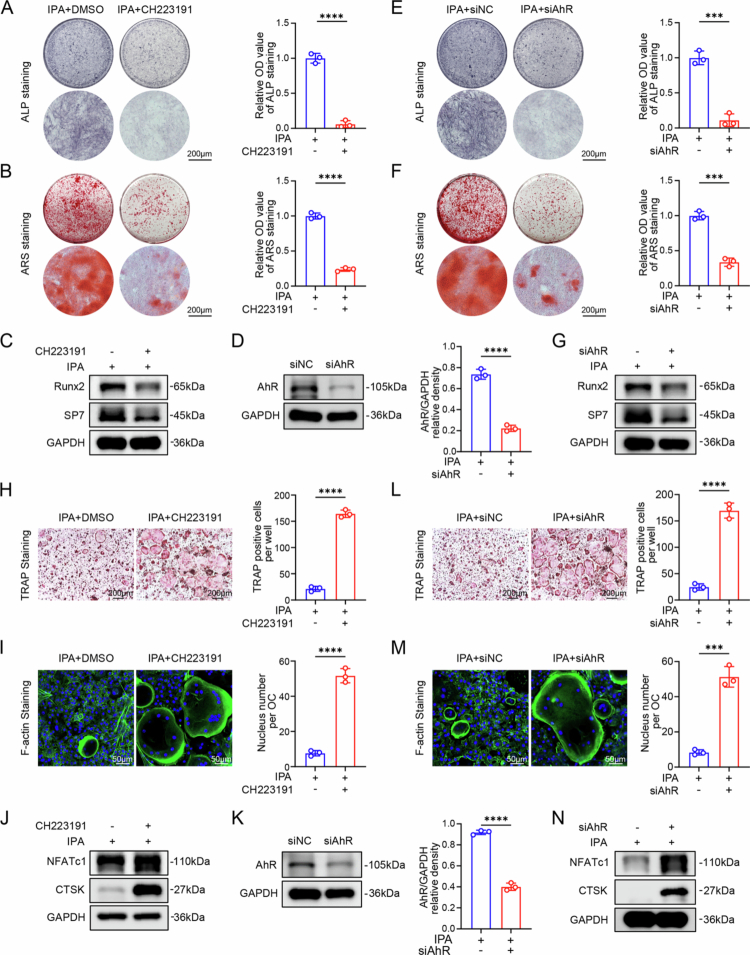
IPA regulates bone metabolism by activating AhR. (A) ALP staining and quantitative analysis of osteoblast differentiation with or without the AhR inhibitor CH223191 (scale bar = 200 µm, *n* = 3). (B) ARS staining and quantitative analysis of osteoblast differentiation with or without CH223191 (scale bar = 200 µm, *n* = 3). (C) Representative western blot images of osteoblast differentiation with or without CH223191. (D) Representative western blot images and quantitative analysis of siAhR knockdown efficiency in BMSCs. (E) ALP staining and quantitative analysis of osteoblast differentiation with or without siAhR (scale bar = 200 µm, *n* = 3). (F) ARS staining and quantification of osteoblast differentiation with or without siAhR (scale bar = 200 µm, *n* = 3). (G) Representative western blot images and quantitative analysis of osteoblast differentiation with or without siAhR. (H) TRAP staining and quantification of osteoclast differentiation with or without CH223191 (scale bar = 200 µm, *n* = 3). (I) F-actin staining and quantification of osteoclast differentiation with or without CH223191 (Scale bar = 50 µm, *n* = 3). (J) Representative western blot images and quantitative analysis of osteoclast differentiation with or without CH223191. (K) Representative western blot images and quantitative analysis of siAhR knockdown efficiency (*n* = 3). (L) TRAP staining and quantification of osteoclast differentiation with or without siAhR (scale bar = 200 µm, *n* = 3). (M) F-actin staining and quantification of osteoclast differentiation with or without siAhR (scale bar = 50 µm, *n* = 3). (N) Representative western blot images and quantitative analysis of osteoclast differentiation with or without siAhR. The data are presented as mean ± SD. Statistical significance was obtained by Student's *t* test (two-tailed). Significance: ***p < 0.001 and ****p < 0.0001.

Given that several indole metabolites signal through AhR, we assessed whether IPA engages this pathway. To determine whether AhR participates in the pro-osteogenic effects of IPA, we utilized the AhR inhibitor CH223191 and constructed an siRNA targeting AhR (siAhR) for osteoblast differentiation. ALP and Alizarin red staining revealed that the ability of IPA effects in promoting osteoblast differentiation and mineralization were significantly reversed following the addition of CH223191 and siAhR (Figure 4A,B,E,F). Western blot analysis showed that the knockdown efficiency of siAhR in primary BMSCs exceeded 50% (Figure 4D). After treatment with CH223191 and siAhR, the protein levels of osteogenic markers (RUNX2 and SP7) were significantly decreased (Figures 4C,G and S11A,B).

To investigate the potential role of AhR in IPA-induced inhibition of osteoclast differentiation, we added CH223191 and siAhR to perform osteoclast differentiation assays. TRAP and F-actin staining revealed that the inhibitory effects of IPA on osteoblast differentiation were notably reversed in the addition of CH223191 and siAhR. This reversal was characterized by an increase in the number of TRAP-positive osteoclasts and the number of nucleus per osteoclast (Figure 4H,I,L,M). Western blot analysis showed that siAhR knockdown efficiency in primary BMMs exceeded 50% (Figure 4K). Following AhR inhibition via CH223191 or siAhR, the levels of osteoclast-associated proteins NFATc1 and CTSK were significantly increased (Figures 4J,N and S11C,D). In conclusion, these findings indicate that IPA exerts dual regulatory effects on bone metabolism by activating AhR, promoting osteoblast differentiation while inhibiting osteoclastogenesis.

### IPA stabilizes β-catenin and IκBα proteins by inhibiting β-TrCP function through AhR activation

**Figure 5.**
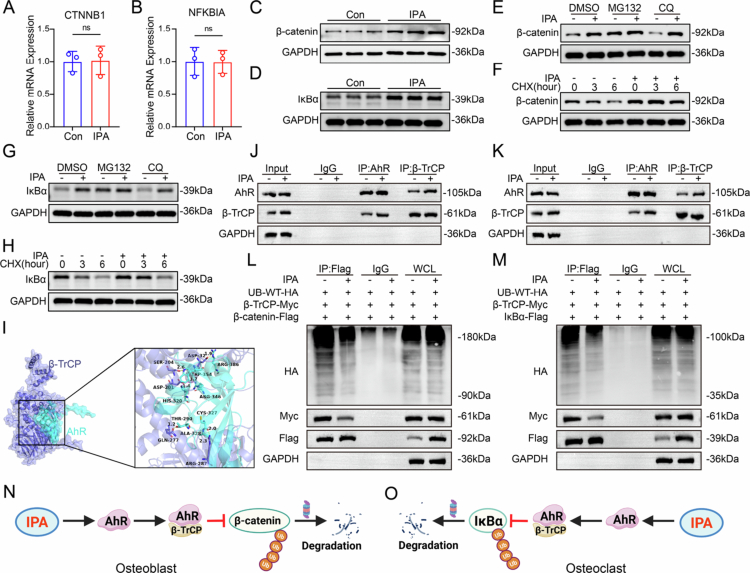
IPA stabilizes β-catenin and IκBα proteins by inhibiting β-TrCP function through AhR activation. (A) qPCR analysis of β-catenin mRNA expression levels before and after IPA (20 μM) treatment (*n* = 3). (B) mRNA expression levels of IκBα before and after IPA (20 μM) treatment (*n* = 3). (C) Western blot analysis of β-catenin protein expression before and after IPA (20 μM) treatment during osteoblast differentiation. (D) Western blot analysis of IκBα protein levels before and after IPA (20 μM) treatment during osteoclast differentiation. (E) Western blot showing the effect of IPA on β-catenin protein levels in MC3T3 cells treated with MG132, CQ, and control (DMSO). (F) Western blot showing the effect of IPA on β-catenin protein levels in MC3T3 cells treated with CHX at the indicated time intervals. (G) Western blot showing the effect of IPA on IκBα protein levels in RAW cells treated with MG132, CQ, and DMSO. (H) Western blot showing the effect of IPA on IκBα protein levels in RAW cells treated with CHX at the indicated time intervals. (I) Protein docking analysis showing the interaction between AHR and β-TrCP. (J and K) Representative immunoprecipitation assays showing the interaction between AHR and β-TrCP in MC3T3 and RAW cells. (L) IPA treatment reduces β-TrCP and ubiquitinated β-catenin levels in the immunoprecipitated (IP) group from MC3T3 cells. (M) IPA treatment reduces β-TrCP and ubiquitinated IκBα levels in the immunoprecipitated (IP) group from RAW cells. (N) The schematic illustrates reflects the underlying mechanisms of IPA in decreasing β-catenin degradation by reducing the level of ubiquitinated β-catenin in MC3T3 cells. (O) The schematic illustrates reflects the underlying mechanisms of IPA in decreasing IκBα degradation by reducing the level of ubiquitinated IκBα in RAW cells. The data are presented as mean ± SD. Statistical significance was obtained by Student's *t* test (two-tailed). Significance: NS, nonsignificance and WCL: whole cell lysate.

Our RNA sequencing results revealed significant enrichment of the Wnt/β-catenin and NF-κB signaling pathways following IPA treatment (Figure 3K,M). To investigate the underlying mechanisms, we then detected mRNA and protein levels of β-catenin (a core component of the Wnt/β-catenin signaling pathway) and IκBα (a key inhibitory component of the NF-κB signaling pathway). Interestingly, the transcription levels of β-catenin and IκBα were not significantly affected by IPA treatment (Figure 5A,B). However, the protein levels were significantly increased (Figures 5C,D and S12A,B), suggesting that IPA may regulates these proteins through post-translational modifications.

Protein degradation through the ubiquitin-proteasome system plays a pivotal role in cellular protein homeostasis and fine-tunes osteoblast and osteoclast differentiation by degrading specific regulatory proteins.^37^^,^^38^ To investigate whether IPA mediates the degradation of β-catenin and IκBα protein, we conducted a series of ubiquitination-related experiments. The proteasome inhibitor MG132, but not the lysosomal inhibitor chloroquine (CQ), prevented the degradation of β-catenin and IκBα, indicating that their degradation is proteasome-dependent (Figures 5E,G and S12C,E). Interestingly, IPA showed a similar effect to MG132, significantly preventing the degradation of these proteins (Figure 5E,G). Further, cycloheximide (CHX)-chase assays revealed that IPA intervention significantly delayed the degradation of these proteins, even in the presence of a protein synthesis inhibitor (Figures 5F,H and S12D,F).

Previously, we demonstrated that IPA regulates bone metabolism by activating AhR. Additionally, we found that inhibiting AhR significantly reduce the protein levels of β-catenin and IκBα (Figure S13A–D). Because β-TrCP serves as the substrate-recognition component of SCF^β-TrCP, which specifically targets IκBα and β-catenin for ubiquitination and proteasomal degradation,^39-43^ we interrogated the AhR–β-TrCP axis. Using molecular docking (Autodock Vina) and protein structure simulations (PyMOL), we found that AhR forms hydrogen bonds with key residues in the β-TrCP active site, stabilizing the AhR-β-TrCP complex with an estimated binding energy of −34.2 kcal/mol (Figure 5I). Further immunoprecipitation experiments demonstrated that AhR and β-TrCP interacted in both the osteoblast precursor cell line (MC3T3) and the monocyte macrophage cell line (RAW) (Figure 5J,K). Notably, the interaction between AhR and β-TrCP increased significantly following IPA treatment (Figure 5J,K). Ubiquitination immunoprecipitation (IP) experiments further confirmed that AhR antagonizes E3 ubiquitin ligase β-TrCP, inhibiting the ubiquitination of IκBα and β-catenin (Figure 5L–O). In summary, these findings demonstrate that IPA inhibits β-TrCP function by activating AhR, stabilizing β-catenin and IκBα proteins, and regulating the Wnt/β-catenin and NF-κB signaling pathways. This dual regulatory mechanism highlights IPA's significant role in modulating bone metabolism.

### Engineered *C. sporogenes* alleviates bone loss in OVX mice

**Figure 6.**
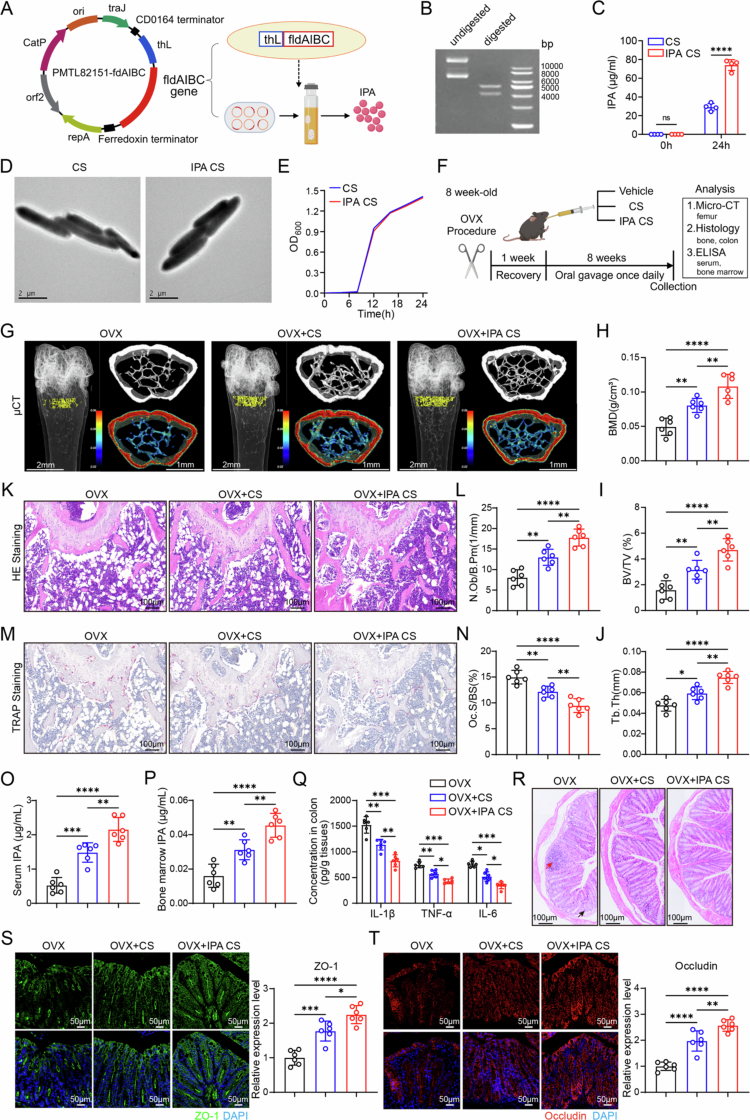
Engineered *C. sporogenes* significantly alleviates bone mass decline and improves intestinal barrier function in OVX mice. (A) Schematic representation of the construction method of engineered *C. sporogenes*. (B) ldentification of the recombinant plasmid “pMTL82151-fldAIBC” with digestion. Lane description (from left to right). Lane 1: undigested. Lane 2: digested with BamHI and XhoI. Lane 3: ladder. (C) IPA content in bacterial supernatants was measured by high-performance liquid chromatography (HPLC) (*n* = 4). (D) Transmission electron microscopy (TEM) images of CS and IPA-CS. (E) Growth curves of CS and IPA-CS at 600 nm absorbance, recorded by a UV spectrophotometer (*n* = 4). (F) Study design of *in vivo* animal experiments. (G) Microcomputed tomography (µCT) 3D reconstruction of mouse femurs (scale bar = 1 mm, *n* = 6). (H–J) Quantitative analysis of the bone structural parameters of the distal femur, including BMD (H), BV/TV (I), and Tb.Th (J) (*n* = 6). (K and L) H&E staining of distal femur slices and quantitative analysis (scale bar = 100 µm, *n* = 6). (M and N) TRAP staining of distal femur slices and quantitative analysis (scale bar = 100 µm, *n* = 6). (O and P) Serum and bone marrow IPA levels in each group (*n* = 6). (Q) Levels of inflammatory cytokines in intestinal tissue (*n* = 6). (R) H&E staining of intestinal tissue: red arrows indicate inflammatory cell infiltration; black arrows show crypt structure disorganization and goblet cell loss (scale bar = 100 µm, *n* = 6). (S and T) Immunofluorescence of ZO-1 and Occludin in colon tissues (scale bar = 50 µm, *n* = 6). The data are presented as mean ± SD. Statistical significance was calculated by Student's *t* test (two-tailed) (C) and one-way ANOVA (H, I, J, L, N, O, P, Q, S, and T). Significance: *p < 0.05; **p < 0.01; ***p < 0.001; ****p < 0.0001; NS, nonsignificance, CS: *Clostridium sporogenes*, and IPA-CS: engineered *Clostridium sporogenes*.

Extensive research has revealed a significant reduction in the abundance of *Clostridia* in both postmenopausal osteoporosis patients and OVX mice.^20^^,^^44^ Among *Clostridia, C. sporogenes* is also decreased in OVX mice*,* accompanied by a decrease in its metabolite IPA, which is detectable in serum.^44^ Furthermore, our aforementioned studies demonstrated that estrogen deficiency compromises gut barrier function, motivating the investigation of probiotic-based therapeutic approaches (Figure 1K–N). As a potential probiotic, *C. sporogenes* metabolizes dietary tryptophan into IPA through the phenyllactate dehydratase gene cluster (fldAIBC), offering a range of beneficial effects.^32^^,^^45^^,^^46^ Therefore, we genetically engineered *C. sporogenes* to enhance its ability to produce IPA (Figure 6A). The double-enzyme digestion electrophoresis of the plasmid indicates that the recombinant plasmid carrying the fldAIBC gene cluster has been successfully constructed (Figure 6B). High-performance liquid chromatography (HPLC) analysis demonstrated that the engineered strain (IPA-CS) significantly increased IPA production compared to unmodified CS at 24 h (Figure 6C). Characterization of IPA-CS revealed no significant differences between IPA-CS and CS in terms of morphology, particle size distribution, zeta potential, or growth curve (Figures 6D,E, S14A,B), indicating that genetic modification did not affect IPA-CS activity. We then constructed an OVX mouse model and administered CS, IPA-CS (1 × 10^9^ CFU/200 μL/mouse, once daily for 8 weeks), or PBS via oral gavage (Figure 6F). After 8 weeks, a micro-CT scan was performed. The results showed that BMD, BV/TV, and Tb.Th were all increased in the IPA-CS-treated OVX mice compared to the OVX control group (Figure 6G–J), indicating that IPA-CS treatment alleviated the bone mass reduction in OVX mice. The μCT result were further corroborated by detailed histological examination of the femur (Figure 6K–N). These results confirmed that IPA-CS significantly alleviated the bone mass decline in OVX mice.

ELISA assays showed that both CS and IPA-CS treatments effectively increased IPA levels in the serum and bone marrow, with a more significant increase effect observed in the IPA-CS-treated mice (Figure 6O,P). Given the established impact of estrogen deficiency on gut function, we assessed the effects of CS and IPA-CS on intestinal barrier integrity. ELISA results for colonic inflammatory cytokines revealed that both treatments reduced IL-1β, TNF-α, and IL-6 levels, with IPA-CS showing greater efficacy (Figure 6Q). Both CS and IPA-CS treatments improved epithelial cell damage and reduced inflammatory cell infiltration in the mucosal layer (Figure 6R). Immunofluorescence staining of colon tissue revealed that the expression of the tight junction proteins ZO-1 and occludin was significantly increased in both the CS and IPA-CS groups, with a more conspicuous observed in the IPA-CS group (Figure 6S,T), indicating enhanced intestinal barrier integrity.

To investigate whether treatment with engineered *C. sporogenes* alters the gut microbiota, we performed 16S rRNA sequencing on fecal samples collected from three experimental mouse groups. Principal coordinates analysis (PCoA) revealed a significant shift in the overall microbial community structure following treatment (ANOSIM, *R* = 0.8195, *p* = 0.001; Figure S15A). While alpha diversity analysis using the Chao1 index indicated an increase in species richness in the CS-treated group, this effect was not observed in the IPA–CS-treated group (Figure S15B), suggesting differential impacts of the treatments on microbial community stability. Importantly, genus-level analysis demonstrated a graded increase in the abundance of *Clostridium* across the groups (Figure S15C,D). To precisely quantify the colonization of the administered strain, we performed TaqMan-based qPCR targeting *C. sporogenes*, which confirmed this graded increase in bacterial abundance (Figure S15E). Furthermore, correlation analysis demonstrated a strong positive relationship between the abundance of *C. sporogenes* and the serum IPA concentration (Figure S15F).

Furthermore, we used primary BMSCs and primary BMMs from the above mouse models for *in vitro* experiments. ALP staining and Alizarin Red staining showed improved bone formation in OVX + CS mice compared to OVX mice and even more significant improvements in OVX + IPA-CS mice (Figure S16A,B). TRAP and F-actin staining demonstrated reduced osteoclast differentiation in OVX + CS mice, with a more pronounced decrease in OVX + IPA-CS mice (Figure S16C,D). Western blot analysis of primary BMSCs demonstrated increased levels of osteogenesis-related proteins (RUNX2 and SP7) and key proteins involved in osteogenesis-related signaling pathways (β-catenin) in the OVX + CS group, with more pronounced increases in the OVX + IPA-CS group (Figure S16E). Conversely, osteoclast differentiation markers (CTSK and NFATc1) were downregulated, and the inhibitory protein IκBα was upregulated in the IPA-CS group compared to both the control and CS groups (Figure S16F). In summary, these findings demonstrate that engineered *C. sporogenes* (IPA-CS) significantly reverses bone loss in OVX mice by promoting osteoblast activity, inhibiting osteoclast differentiation, and enhancing intestinal barrier function as well as modulating the gut microbiota. Additionally, histological evaluation of major organs in IPA-CS-treated mice demonstrated excellent safety and tolerability, with no significant toxicity or adverse effects observed (Figure S17A). This study highlights the potential of IPA-CS as a probiotic therapeutic approach for osteoporosis.

## Discussion

IPA has increasingly been recognized as a pleiotropic microbiota-derived metabolite that, in addition to its barrier-protective and immunomodulatory roles, also exerts neuroprotective and metabolic regulatory functions.^15^^,^^16^^,^^47^^,^^48^ Our study sheds light on a previously insufficiently characterized association between IPA and PMOP, identifying IPA as a metabolic regulator of bone homeostasis that acts by coordinating the coupling of bone formation and bone resorption. Mechanistically, IPA activates AhR to inhibit the E3 ubiquitin ligase β-TrCP, thereby stabilizing β-catenin and IκBα proteins and modulating the function of Wnt/β-catenin and NF-κB signaling pathways, ultimately exerting a dual regulatory effect on bone metabolism. Although prior studies have implicated AhR in bone biology,^22^ its downstream signaling is known to be ligand- and cell type-dependent.^49^ For example, AhR agonists such as TCDD and FICZ suppress mesenchymal stem cell osteogenic differentiation while promoting osteoclastogenesis,^50^^,^^51^ whereas other ligands like BaP have been reported to inhibit osteoclast formation and bone resorption.^52^ Within this heterogeneous landscape, our work specifically connects the microbiota-derived metabolite IPA to AhR activation and proposes an IPA-driven AhR–β-TrCP–β-catenin/IκBα axis in the regulation of bone remodeling. Nevertheless, we acknowledge that sustained or context-dependent activation of AhR may pose potential risks, including the engagement of oncogenic pathways, which remains an important issue to be considered in future studies.^53^

Engineered probiotics have been applied in various therapeutic contexts, including skeletal disorders.^54^ Engineering modifications of *C. sporogenes* to enhance the production of specific metabolites have been applied in contexts such as cancer therapy and bile acid dihydroxylation, underscoring its suitability as a metabolite-producing chassis.^55^^,^^56^ In contrast, commonly used engineered probiotics—including *E. coli* Nissle 1917 and various *Lactobacillus* strains—have been broadly applied for therapeutic compounds delivery but do not natively synthesize IPA or possess the endogenous enzymatic machinery required for its production.^57^^,^^58^ Based on this distinction, we leveraged the intrinsic ability of *C. sporogenes* to convert dietary tryptophan into IPA and constructed an engineered strain (IPA-CS) carrying a plasmid-borne fldAIBC gene cluster to enhance its biosynthetic capacity. *In vivo*, IPA-CS effectively colonized the gut and was associated with attenuation of OVX-induced bone loss, together with reduced intestinal inflammation and improved barrier integrity. Together, these findings deepen the understanding of PMOP pathophysiology and provide a conceptual basis for developing microbiota-based therapeutic approaches.

To evaluate the functional relevance of IPA *in vivo*, we initially administered intraperitoneal IPA, which confirmed its osteoprotective effect in OVX mice. However, repeated systemic injections are invasive and generate transient pharmacokinetic peaks that do not reflect the sustained luminal production of IPA by gut microbes.^59^ Moreover, IPA displays high plasma protein binding and low free drug levels in humans, complicating systemic bioavailability when delivered by bolus dosing.^60^ Although several studies have assessed orally administered IPA and reported protective effects in other injury settings,^61^^,^^62^ its absorption is variable and may not reliably re-establish luminal IPA concentrations, especially under conditions of impaired barrier integrity.^63^ These limitations motivated the use of an engineered *C. sporogenes* approach to enhance in situ IPA biosynthesis, which is conceptually aligned with existing live biotherapeutic designs.^64^^,^^65^ Furthermore, beyond PMOP, dysregulation of IPA and microbiota-derived indole–AhR signaling has been linked to other chronic disorders affecting bone and systemic metabolism, including inflammatory arthritis and cancer cachexia.^18^^,^^66^ On this basis, we cautiously speculate that engineered *C. sporogenes* strains may, in the future, be explored as a conceptual approach for these additional disorders, although such possibilities remain highly preliminary and would require extensive validation before any therapeutic application can be considered.

Several limitations should be acknowledged. First, the relatively small sample size, together with the fact that serum IPA levels can be substantially influenced by dietary composition, microbiota diversity, and host genetic variation, may limit the generalizability of the findings and contribute to interindividual variability in IPA responses. Although the study was sufficiently powered to detect statistically significant differences in prespecified endpoints, larger and prospectively designed cohorts will be required to validate these observations across more heterogeneous populations. Second, although the OVX mouse model captures major features of estrogen deficiency–induced bone loss,^67^ it does not fully recapitulate the gradual, multifactorial progression of human PMOP, underscoring the need for validation in complementary preclinical models and eventually in human studies. Finally, although no overt toxicity or adverse effects were observed in mice, engineering *C. sporogenes* raises additional safety, genetic stability, colonization dynamics, and regulatory concerns that will require further evaluation, particularly in the context of potential human translation.

Future studies should further refine our understanding of the gut–bone axis and delineate how microbial metabolites integrate with host signaling networks to regulate skeletal homeostasis. Beyond IPA, comprehensive profiling of microbial tryptophan metabolites and other gut-derived small molecules may reveal additional factors that contribute to bone remodeling. Furthermore, extending investigation beyond estrogen deficiency–induced bone loss to other skeletal conditions—such as fracture healing, critical-sized bone defects, and prosthesis-associated osteolysis—may help clarify the breadth and boundaries of microbiota-derived metabolic regulation in diverse orthopedic contexts. Collectively, these future directions will advance mechanistic insight into the microbiota–bone axis and inform the rational development of targeted microbial or metabolite-based strategies.

Overall, we sought to determine whether dysregulated tryptophan metabolism and reduced IPA levels may contribute to the pathogenesis of PMOP, explored the potential mechanisms involved, and designed an engineered *C. sporogenes* strain as a potential approach for alleviating postmenopausal bone loss.

## Materials and methods

### Ethical approval

This study adhered to all relevant ethical regulations and was approved by the Ethics Committee of Soochow University (ethics approval number: SUDA20241210A04) and the Second Affiliated Hospital of Soochow University (ethics approval number: JD-LK2024098-IR01).

### Materials

The antibodies applied in this study were shown in Supplementary Table 1.

### Mice and reagents

Animal experiments were approved by Soochow University Laboratory Animal Centre (approval number SUDA20241210A04). Female C57BL/6J mice, aged 6–8 weeks, were obtained from the Laboratory Animal Center of Soochow University. The mice were housed in ventilated cages under specific pathogen-free (SPF) conditions and maintained between 22 °C and 24 °C and a controlled 12-h light‒dark cycle. The mice had ad libitum access to drinking water and standard laboratory chow, ensuring their good physiological conditions. α-MEM, DMEM, fetal bovine serum (FBS), streptomycin, and penicillin for cell culture were purchased from Gibco BRL (Grand Island, NY, USA). Recombinant mouse macrophage colony-stimulating factor (M-CSF) and receptor activator of nuclear factor-κB ligand (RANKL) were obtained from R&D Systems (Minneapolis, MN, USA). IPA was purchased from Aladdin (I103959), while CH223191 (HY-12684), MG132 (HY-13259), chloroquine (CQ) (HY-17589A), and cycloheximide (CHX) (HY-12320) were obtained from MedChemExpress (NJ, USA).

### Population information

Serum samples were collected from normal laboratory bone mass women (BMD T-score ≥−1.0) and PMOP patients (BMD T-score ≤−2.5) after obtaining informed consent. The study protocol was approved by the Ethics Committee of the Second Affiliated Hospital of Soochow University (ethics approval number: JD-LK2024098-IR01) and conducted in strict accordance with the relevant guidelines. General demographic information, including age, height, weight, and underlying diseases, were recorded, and bone mineral density (BMD) was measured at the proximal femur using dual-energy X-ray absorptiometry (GE Medical Systems, Lunar). There was no significant difference in the baseline data between the two groups of patients. Detailed population clinical characteristics are provided in Supplementary Table 2. Notably, β-CTX levels were significantly elevated in the PMOP group compared with the Normal group.

Inclusion criteria: all participants were postmenopausal women recruited through our osteoporosis clinic who met the following inclusion criteria: (1) natural menopause for at least 5 y, (2) proximal femur bone mineral density (BMD) measured by dual-energy X-ray absorptiometry (DXA) with T-scores either ≤−2.5 (for the PMOP group) or ≥−1.0 (for the normal group), and (3) willingness to provide informed consent for study participation.

Exclusion criteria: (1) any condition known to cause secondary osteoporosis (including severe diabetes mellitus, primary hyperparathyroidism, rheumatoid arthritis, or multiple myeloma); (2) history of spinal surgery, spinal tumors, ankylosing spondylitis, or diffuse idiopathic skeletal hyperostosis; (3) current or recent (within 6 months) use of medications affecting bone metabolism (including bisphosphonates, SERMs, denosumab, parathyroid hormone analogs, romosozumab, and hormone replacement therapy); (4) history of cancer requiring chemotherapy; or (5) active gastrointestinal disorders or current proton pump inhibitor use that might influence nutrient absorption and bone metabolism.

### Untargeted metabolomics analysis

A 100 μL aliquot of serum was transferred to a 1.5 mL microcentrifuge tube, after which 400 μL of pre-cooled extraction solvent (acetonitrile: methanol, 1:1, v:v) containing the internal standard L-2-chlorophenylalanine (0.02 mg mL⁻¹) was added. The mixture was vortex-mixed for 30 s, sonicated for 30 min at 5 °C (40 kHz), and then chilled at −20 °C for a further 30 min to precipitate the proteins. The samples were then centrifuged at 13,000 × *g* for 15 min (4 °C). The resulting supernatant was evaporated to dryness under a gentle stream of nitrogen. The residues were redissolved in 100 μL of acetonitrile:water (1:1, v:v), resonicated for 5 min under the same low-temperature conditions, and clarified again by centrifugation (13,000 *g*, 10 min, 4 °C). The final supernatant was transferred to autosampler vials for LC–MS analysis.

Metabolomic data were acquired on a Thermo Scientific UHPLC-Q Exactive system at Majorbio BioPharm Technology Co. Ltd. (Shanghai, China). Mobile phase A consisted of 95% H₂O + 5% acetonitrile with 0.1% formic acid, while mobile phase B comprised 47.5% acetonitrile + 47.5% isopropanol + 5% H₂O, also containing 0.1% formic acid. Electrospray ionization was operated in both positive and negative modes over an m/z range of 70–1050. The instrument parameters were: sheath gas 50 psi, auxiliary gas 13 psi, auxiliary gas heater 425 °C; spray voltage + 3.5 kV (ESI⁺)/−3.5 kV (ESI^−^), ion-transfer tube 325 °C, and stepped collision energy 20/40/60 eV. Full-scan spectra were collected at 60,000 resolution, and data-dependent MS/MS spectra at 7500 resolutions.

The UHPLC-MS raw data were processed using Progenesis QI software for baseline correction, peak alignment, and feature extraction, generating a comprehensive data matrix of mass-to-charge ratios, retention times and normalized intensities. Metabolite identification was performed by querying the HMDB, Metlin and in-house MJDB databases. Subsequent data preprocessing on the Majorbio platform involved: (1) retaining metabolic features detected in ≥80% of samples per experimental group, (2) imputing missing values using minimum observed intensities, and (3) applying sum normalization to correct for technical variation. Following quality control filtration (excluding variables with >30% RSD in QC samples) and log10 transformation, multivariate statistical analysis was conducted using the ropls package (v1.6.2), incorporating both PCA and OPLS-DA with 7-cycle cross-validation to identify significantly altered metabolites (VIP > 1 from OPLS-DA and *p* < 0.05 by Student's *t* test). Metabolites were annotated against the HMDB (http://www.hmdb.ca/) and Metlin (https://metlin.scripps.edu/) databases, and differentially abundant compounds were mapped to pathways using the KEGG resource.

### Targeted tryptophan metabolomic analysis

Accurately weigh 33 standard substances related to the tryptophan metabolic pathway and prepare individual stock solutions using 50% methanol. Combine the standards to create a mixed standard solution and dilute it with ultrapure water to achieve a range of concentrations. Accurately weigh the appropriate amount of the isotopic standard (Trp-D5), prepare a methanol-based stock solution, and dilute it with ultrapure water to a concentration of 4000 ng/mL for the isotopic internal standard working solution.

Accurately transfer 100 μL of the serum sample to a tube, and add 10 μL of the isotopic internal standard working solution (Trp-D5, 4000 ng/mL). Then, add 390 μL of methanol as the extraction solvent, followed by vortex mix for 30 s. Subject the mixture to cryogenic ultrasonication for 30 min (5 °C, 40 kHz), followed by standing at −20 °C for 30 min. Centrifuge the mixture at 4 °C and 13,000 × *g* for 15 min. Collect 400 μL of the supernatant and dry it under nitrogen. Reconstitute with 100 μL of 1% acetonitrile in water (containing 0.1% formic acid), vortex mix for 30 s, and perform cryogenic ultrasonication again for 15 min (5 °C, 40 kHz). Centrifuge again at 4 °C, 13,000 × *g* for 15 min, and collect the supernatant for analysis.

LC‒MS/MS analysis was performed using a Nexera Series LC-40 liquid chromatography system combined with a QTRAP® 6500+ mass spectrometer (Sciex, USA) at Majorbio Bio-Pharm Technology Co. Ltd. (Shanghai, China). A 2 μL aliquot sample was injected and separated on an ACQUITY UPLC HSS T3 column (2.1 × 150 mm, 1.8 μm) for mass spectrometry analysis. The mobile phase consisted of 0.1% formic acid in water (Phase A), and 0.1% formic acid in acetonitrile (Phase B). The separation gradient was as follows: 0.0–2.5 min, 1%–11% B; 2.5–5.5 min, maintain 11% B; 5.5–6.5 min, 11%–28% B; 6.5–7.5 min, maintain 28% B; 7.5–12.5 min, 28%–50% B; 12.5–13.5 min, 50%–95% B; 13.5–15.5 min, maintain 95% B; 15.5–15.6 min, 95%–1% B; and 15.6–18 min, maintain 1% B. The flow rate was set at 0.35 mL/min, with the column temperature maintained at 40 °C.

The LC–MS raw data were imported into Sciex OS quantitation software. Using default parameters, the software automatically identified and integrated each ion fragment, with manual inspection conducted for verification. The final concentrations of the analytes were calculated by substituting their mass spectrometry peak areas into the respective standard linear equations.

### Cell proliferation and viability

The cytotoxicity effects of IPA on osteoclasts and osteoblast progenitors were evaluated using the Cell Counting Kit-8 (CCK-8) assay (MA0218, Meilunbio). BMMs, RAW264.7 cells, BMSCs, and MC3T3-E1 cells were seeded into 96-well plates at a density of 5 × 10³ cells/well. IPA was added at the indicated concentrations, and the cells were incubated for 24, 48, and 72 h. After incubation, the culture medium was replaced with fresh medium containing 10% CCK-8, and the cells were incubated for an additional hour at 37 °C. The absorbance was measured at 450 nm using a BioTek microplate reader (BioTek, Winooski, VT, USA). Cell viability was calculated by comparing the absorbance values with those of the untreated control.

### Osteoblast differentiation, ALP staining, and mineralization assay

For the osteoblast culture and differentiation experiments, BMSCs were isolated from 6- to 8-week-old C57BL/6 female mice. The cells were seeded in α-MEM supplemented with 10% fetal bovine serum (FBS) and 1% penicillin–streptomycin and cultured at 37 °C in a humidified atmosphere with 5% CO_2_. To induce osteogenic differentiation, BMSCs were plated in 12-well plates at a density of 1 × 10⁴ cells per well and treated with an osteogenic induction medium containing β-glycerophosphate (10 mM), ascorbic acid (50 μg/mL), and dexamethasone (10 nM). When appropriate, IPA was added as an intervention. The culture medium was replaced every 3 d. After 7 d of osteoblast induction, ALP staining assay was performed according to the instructions of the alkaline phosphatase staining kit (#C3206, Beyotime, Beijing, China). On day 14, Alizarin Red S (ARS) staining was performed using a calcium staining kit following a modified Alizarin Red S method (Solarbio, Beijing, China) to assess matrix mineralization. The stained areas were visualized using microscopy, and image quantification was performed with ImageJ software.

### Osteoclast differentiation, TRAP staining, and F-actin staining assay

Bone marrow cells were obtained by gently perfusing the femora and tibiae of the mice with α-MEM and then suspended in α-MEM supplemented with 10% fetal bovine serum (FBS) and 1% penicillin–streptomycin. The suspension was incubated overnight at 37 °C in 5% CO₂ to permit stromal adherence. The following day, nonadherent cells were transferred to fresh medium containing 30 ng mL⁻¹ macrophage colony-stimulating factor (M-CSF) and cultured for 3 d to generate bone marrow–derived macrophages (BMMs). For osteoclastogenesis, BMMs were plated at 1 × 10⁴ cells per well in 96-well plates and maintained for 5–7 d in α-MEM supplemented with 30 ng mL⁻¹ M-CSF and 50 ng mL⁻¹ receptor activator of nuclear factor-κB ligand (RANKL). Tartrate-resistant acid phosphatase (TRAP) staining kit (387A-1KT, Sigma) was performed after fixation with 4% paraformaldehyde for 10 min, cells were developed at 37 °C for 1 h, and TRAP-positive multinucleated cells (≥3 nuclei) were counted under an Olympus microscope (Waltham, MA, USA).^44^ For F-actin visualization, the fixed cultures were permeabilized with 0.1% Triton X-100 for 10 min, stained for 30 min at room temperature with FITC-conjugated phalloidin (Solarbio, Beijing, China), and counterstained with DAPI (Beyotime, Shanghai, China). Confocal images were collected, and ImageJ was used to quantify the number of nuclei per osteoclast.

### Real-time quantitative PCR (qPCR)

Cellular RNA was isolated from cultured cell lines employing TRIzol reagent (Takara, Japan) following the supplier's recommended procedures. cDNA synthesis was subsequently performed with HiScript III All-in-one RT SuperMix (Vazyme, Nanjing, China). Quantitative PCR was carried out on a CFX Connect Real-Time PCR instrument using HiScript II Q RT SuperMix (Vazyme, Nanjing, China), with glyceraldehyde-3-phosphate dehydrogenase (GAPDH) serving as the internal control for data normalization.^44^ The corresponding primer sequences have been compiled in Supplementary Table 3.

### Western blot

Following a PBS rinse, the cells were lysed on ice in RIPA buffer supplemented with 1% phosphatase and 1% protease inhibitor cocktails (Beyotime, China). The homogenate was clarified by centrifugation at 12,000 × *g* for 15 min, and the protein content in the resulting supernatant was quantified using the Beyotime BCA assay. Equal protein loads were then separated by SDS‒PAGE and electrotransferred onto polyvinylidene difluoride (PVDF) membranes. After blocking for 1 h with 5% skim milk, the membranes were incubated overnight at 4 °C with the appropriate primary antibodies. Horseradish peroxidase–conjugated secondary antibodies were subsequently applied, and signals were developed with enhanced chemiluminescence (ECL).^68^ Densitometric analysis of band intensity was performed with ImageJ software.

### Co-IP (co-immunoprecipitation) assay

After transfection or stimulation, the cells were lysed with NP40 lysis buffer (#P0013, Beyotime, China) containing 1% phosphatase inhibitor and 1% protease inhibitor for 30 min on ice. The lysates were then centrifuged at 12,000 rpm for 5 min at 4 °C. For immunoprecipitation, the cell lysates were incubated with a mixture of exogenous tag protein antigen and antibody, and 30 μL of Flag/Myc/HA antibody-conjugated agarose beads were added. The lysates (without protease inhibitors) were washed once, combined with the antigen‒antibody mixture, and incubated overnight in a cold chamber. The next day, the mixture was centrifuged at 1000 × *g* for 1 min at 4 °C, and the agarose bead-antibody-antigen complex was collected. The supernatant was discarded, and the beads were washed 3× with 500 μL of precooled NP40 buffer. Finally, the agarose bead‒antigen‒antibody complexes were resuspended in 60 μL of loading buffer, denatured at 100 °C for 5 min, and analyzed by western blotting.

### AhR block and transfection

A 2 mL suspension containing 5.0 × 10⁵ BMMs per well was seeded in 6-well plates and incubated for 24 h. Transfection of 10 nM siRNA (Gemma Gene, China) was performed using Lipofectamine 3000 reagent (Thermo Fisher Scientific) according to the manufacturer's protocol. After 8 h, the medium was replaced with osteoclast induction medium containing M-CSF (30 ng/mL) and RANKL (100 ng/mL) to induce the formation of mature osteoclasts. Total RNA was collected 24–48 h after the medium was changed, and total protein was collected 48–72 h post-treatment. For osteoblasts differentiation using BMSCs, transfection was typically performed on days 2 and 4, with RNA samples collected on day 5 for further analysis, protein samples were collected on day 7. siAhR sequences: Sense: 5′-GCGUAUAUGAGCUCAUCCATT-3′, Antisense: 5′- UGGAUGAGCUCAUAUACGCTT-3′.

### MG132 and the CHX experiments

To investigate the effect of IPA on protein degradation pathways, cells were treated with the proteasome inhibitor MG132 (10 μM), the lysosomal inhibitor chloroquine (CQ, 20 μM), or DMSO as a control. After the designated treatment period, total cellular proteins were extracted and analyzed by western blotting. After treatment with the protein synthesis inhibitor cycloheximide (CHX, 100 μg/mL) for 0, 3, and 6 h, total cellular proteins were extracted and analyzed by western blotting.

### Molecular docking

The X-ray crystal structure of AHR (PDB ID: 8H77) was obtained from the Protein Data Bank (https://www2.rcsb.org/), while the predicted structure of BETA-TRCP was generated using Alphafold. Structural optimizations, including manual water removal and hydrogenation, were performed using AutoDockTools-1.5.7.^69^ Protein‒protein docking was carried out using the GRAMM docking server.^70^ The resulting protein‒protein complexes were further manually optimized by removing water molecules and adding hydrogen atoms. Protein interaction predictions and the generation of protein‒protein interaction maps were conducted using PyMOL. In the visualizations, BETA-TRCP is represented as a dark blue cartoon model, while AHR is shown as a cyan cartoon model. Their interaction sites are depicted as stick structures in corresponding colors. Multiple residues contribute to the formation of hydrogen bonds between BETA-TRCP and AHR, such as the bond between ASP32 of BETA-TRCP and ARG386 of AHR. These interactions resulted in a BETA-TRCP-AHR docking hit score of −549, indicating a strong interaction. Further analysis using PDBePISA (https://www.ebi.ac.uk/msd-srv/prot_int/pistart.html) revealed that the calculated binding energy between BETA-TRCP and AHR is –34.2 kcal/mol.

### RNA sequencing and analysis

MC3T3-E1 cells were cultured in osteogenic medium for 3 d in the presence or absence of IPA, and BMMs were cultured in osteoclast induction medium for 3 d, all in 6-well plates. Total RNA was extracted from the harvested cells using TRIzol reagent and assessed using an Agilent 5300 Bioanalyzer (Agilent, USA). RNA sequencing libraries were prepared using the Illumina NovaSeq Reagent Kit and sequenced on the Illumina NovaSeq Xplus platform (Illumina, San Diego, CA, USA). Low-quality reads and adapter sequences were removed from the raw reads. The clean data were then aligned to the reference genome after quality control. Gene and transcript expression levels were quantified using RSEM software. Differentially expressed genes between the two groups were identified with DESeq2 software. A fold change greater than 2 and a *p*-value < 0.05 were considered statistically significant. Gene Ontology (GO) and KEGG pathway enrichment analyses were subsequently performed on the identified DEGs to explore their functional relevance.

### Engineered *C. sporogenes*

The methodology adopted for constructing the engineered probiotic is consistent with that reported in our previous study.^68^ To generate a *Clostridium sporogenes* strain capable of high-level indole-3-propionic acid (IPA) synthesis, the *Clostridium*–*E. coli* shuttle plasmid pMTL82151 was selected as the backbone. The strong promoter Pthl was PCR-amplified with the primers thl-XbaI-F/R, digested with XbaI and XhoI, and ligated into identically digested pMTL82151, creating pMTL82151-thl. A chemically synthesized fragment encompassing the fldAIBC operon—the structural genes of the *E. coli* IPA pathway—was then excised with matching restriction sites and inserted downstream of Pthl, yielding the expression construct pMTL82151-fldAIBC. The recombinant plasmid was heat-shock transformed into *E. coli*; chloramphenicol-resistant colonies were expanded in LB containing chloramphenicol. For conjugal transfer, the *E. coli* donor carrying pMTL82151-fldAIBC was mixed with *C. sporogenes* grown in reinforced clostridial medium (RCM), plated on RCM, and incubated anaerobically at 37 °C. After mating, the cells were recovered and spread on RCM supplemented with chloramphenicol (15 µg ml⁻¹) to select the engineered IPA-producing probiotic.

The morphology, hydrodynamic diameter, and zeta potential of the wild-type and engineered bacteria were examined by transmission-electron microscopy (FEI TF20) and dynamic light scattering analysis (Nano ZS90, Malvern). Growth kinetics were evaluated by culturing both strains statically at 37 °C in RCM with or without 15 µg ml⁻¹ chloramphenicol and recording the absorbance at 600 nm at predefined intervals. For metabolite determination, −80 °C glycerol stocks of the engineered strain were revived anaerobically in RCM containing chloramphenicol (15 µg ml⁻¹) and incubated for 24 h at 37 °C. Cultures were clarified by centrifugation (12,000 rpm, 10 min), and the supernatants were passed through 0.2 µm filters. IPA concentrations were quantified on an HPLC system (Thermo UltiMate 3000) under isocratic elution with 20% acetonitrile and 80% 0.1% trifluoroacetic acid for 25 min at 1.0 ml min⁻¹, with detection at 280 nm. IPA levels (µg ml⁻¹) were calculated by comparing peak heights or areas with those of a certified IPA standard (Yuanye, Shanghai, China).

### 16S rRNA sequencing

Fecal samples were subjected to microbial community analysis by Shanghai Majorbio Bio-Pharm Technology Co. Ltd. (Shanghai, China). Genomic DNA was isolated using the E.Z.N.A.® Soil DNA Kit (Omega Biotek, Norcross, GA, USA) in accordance with the manufacturer's protocol. DNA purity and concentration were determined with a NanoDrop spectrophotometer (Thermo Scientific, USA), and integrity was confirmed by agarose gel electrophoresis. The bacterial 16S rRNA gene covering the V4–V5 hypervariable regions was amplified using primers 338F and 806R. PCR products were excised from 2% agarose gels, purified with the AxyPrep DNA Gel Extraction Kit (Axygen Biosciences, CA, USA), and quantified by QuantiFluor™-ST (Promega, USA). Equal amounts of purified amplicons were pooled and sequenced on the Illumina MiSeq platform using a paired-end 2 × 300 bp strategy.

Raw FASTQ data were demultiplexed with an in-house Perl script. Quality control was performed using fastp (v0.19.6), and paired-end reads were merged with FLASH (v1.2.7) under the following conditions: (1) reads were truncated if the mean quality score dropped below 20 in a 50 bp sliding window, and any read shorter than 50 bp or containing ambiguous bases was removed; (2) only overlapping sequences longer than 10 bp were retained, allowing a maximum mismatch rate of 0.2 in the overlap; (3) reads were assigned to samples based on exact barcode matching, allowing up to two mismatches in primer recognition, and sequence orientation was corrected accordingly. High-quality sequences were denoised and clustered into amplicon sequence variants (ASVs) using UPARSE (v7.1), with the most abundant sequence in each variant selected as its representative. To normalize sequencing depth, reads were rarefied to 20,000 per sample, resulting in an average Good's coverage of 99.09%.

### TaqMan real-time PCR

Quantitative detection of *C. sporogenes* was conducted using a TaqMan-based real-time PCR approach. The PP-BioMole-083 universal assay was applied in accordance with the manufacturer's protocol. The primers and probe used were as follows: forward primer 5′-TTAATACCGCATAACATAAGAGAA-3′, reverse primer 5′-CCAGAAAACAGGGCTTTAC-3′, and probe FAM-ATTGCTTTGAGATGGACCCGCG-LFN. The reactions were run on an ABI PRISM 7500 detection platform (Applied Biosystems, USA). The amplification profile began with incubation at 50 °C for 2 min to activate uracil-N-glycosylase, followed by denaturation at 95 °C for 2 min. This was succeeded by 10 cycles of 95 °C for 15 s, 55 °C for 30 s, and 72 °C for 30 s, and then 35 cycles of 95 °C for 15 s and 60 °C for 1 min.

### Animal models

Eight-week-old female C57BL/6 mice were randomly assigned to four groups: Sham, Sham + IPA, OVX, and OVX + IPA. The mice in the OVX and OVX + IPA groups underwent bilateral ovariectomy. Beginning one week post-surgery, the mice in the Sham + IPA and OVX + IPA groups received intraperitoneal injections of IPA (20 mg/kg; the dosage and administration route were based on established literature^15^^,^^19^^,^^47^^,^^71^), while control groups were administered an equivalent volume of PBS three times weekly. After 8 weeks, serum and bone tissue samples were collected for further analysis.

For bacterial treatment, the OVX mouse model was constructed. The experimental group received engineered *Clostridium sporogenes* (1 × 10⁹ CFU/200 μL/mouse) once daily via oral gavage for 8 weeks, while the control group received an equivalent dose of wild-type *C. sporogenes*. After 8 weeks of treatment, serum and bone tissue samples were collected for subsequent experiments.

### μCT analysis

Micro-CT scanning and analysis were performed at Pingsheng Medical Technology (Kunshan). Mouse femurs were scanned using a NEMO MicroCT system (Model NMC-100). The samples were positioned between the X-ray source and the CMOS detector. During scanning, the samples were rotated 360° along the vertical axis for projection imaging. Images were captured by the CMOS detector and processed using image analysis software. The scanning parameters included a voltage of 90 kV, a current of 0.04 mA, and a total scan time of 20 min. Image reconstruction was performed using Avatar software with the Feldkamp–Davis–Kress (FDK) algorithm at a pixel size of 0.012 mm. The static parameters used for quantitative assessment included bone mineral density (BMD), bone volume fraction (BV/TV), bone surface area-to-tissue volume ratio (BS/TV), trabecular thickness (Tb.Th), and trabecular number (Tb.N).

### Histomorphological analysis

For bone histomorphological analysis, femoral specimens were fixed in 4% paraformaldehyde (PFA) for 48 h, followed by decalcification in 15% ethylenediaminetetraacetic acid (EDTA) for 2 weeks, and then embedded in paraffin. 5 μm thick sections were prepared and stained with tartrate-resistant acid phosphatase (TRAP) and hematoxylin and eosin (H&E). Bone tissue parameters were quantified using OsteoMeasure software (OsteoMetrics Inc., Decatur, GA, USA).

For colon histomorphological analysis, 5 μm thick sections of colon tissue were stained with H&E. Additionally, H&E staining was performed on major organs, including the heart, liver, spleen, lungs, and kidneys, to assess the biosafety of IPA treatment.

### Immunofluorescence staining

For immunofluorescence, paraffin sections were deparaffinized in xylene and rehydrated through a graded alcohol series. Antigen retrieval was performed in Tris-EDTA buffer (pH 9.0) (Solarbio, Beijing, China) or 10 mM citrate buffer (pH 9.0) (Solarbio, Beijing, China) for 20 min. Then, the paraffin slices were permeabilized with 0.3% (v/v) Triton X-100 (Sangon, Shanghai, China) for 10 min and blocked with 10% (v/v) goat serum (Beyotime, Shanghai, China) for 1 h. The primary antibodies were incubated at 4 °C overnight. After three washes with PBST, the appropriate secondary antibodies (Servicebio) were incubated for 1 h in the dark, followed by nuclear staining with DAPI (Boster Biological). The colonic barrier integrity was assessed using ZO-1 (GB12195, Servicebio) and Occludin (GB111401, Servicebio) antibodies, while immunofluorescence staining of bone tissue sections was performed using osteoblast-specific proteins such as Sp7 (GB111900, Servicebio), as well as osteoclast-specific proteins CTSK (GB111276, Servicebio). Images were captured using an LSM 800 confocal microscope (Zeiss, Germany), and protein expression levels were quantitatively analyzed using ImageJ software to evaluate bone tissue activity and differentiation under various experimental conditions.

### Enzyme-linked immunosorbent assay (ELISA)

The concentrations of specific markers in the serum, bone marrow, and colon tissue were quantified using enzyme-linked immunosorbent assay (ELISA). Blood samples were collected from the mice, allowed to clot at room temperature for 30 min, and then centrifuged at 3500 × *g* for 10 min. After centrifugation, the serum was aliquoted and stored at −80 °C for subsequent analysis. IPA concentrations in the serum and bone marrow were measured according to the instructions provided with the ELISA kit (#CB11098-Mu, COIBO BIO, China). Additionally, inflammatory markers in colon tissue, including IL-1β, TNF-α, and IL-6, were quantified using ELISA kits from MULTI SCIENCES (USA) following the manufacturer's protocol.

### Statistical analysis

All the results were expressed as mean ± SD. Unless otherwise stated, all the experiments used biological replication. For comparisons between two groups, a two-tailed Student's *t* test was performed, and if the data were not normally distributed, the nonparametric test was used. One-way ANOVA using the Tukey post-test for more than two groups compared. All the statistical analyses were performed using GraphPad Prism (9.0). The results were significant when ****p < 0.0001; ***p < 0.001; **p < 0.01; and *p < 0.05. All experiments were run at least in triplicate.

## Disclosure of potential conflicts of interest

No potential conflicts of interest were disclosed.

## Supplementary Material

Supplementary material Supplementary Materials.docx

## Data Availability

The authors state that all additional data supporting the results of this study are available in the paper or in supplementary information files.
