# Supplementary material for: Engineered probiotic-derived indole-3-propionic acid inhibits ubiquitination via AHR signaling to treat postmenopausal osteoporosis
Source: Gut Microbes. 2026 Jan 8;18(1):2612620. doi: 10.1080/19490976.2025.2612620 (PMC12795275; doi:10.1080/19490976.2025.2612620)
Supplement: Supplementary material — s.docx [file KGMI_A_2612620_SM0042.docx]

**Engineered Probiotic-Derived Indole-3-Propionic Acid Inhibits Ubiquitination via AHR Signaling to Treat Postmenopausal Osteoporosis**

Xueli Qiu^1#^, Lijun Wu^2#^, Fengxian Jiang^1^, Huajian Shan^1^, Lei Sheng^1^, Bo Tian^1^, Heng Wang^2^, Hao Cui^1^, Lide Tao^1^, Chenyang Wu^1^, Yuqian Yao^1^, Chao Wang^3^*, Xiaozhong Zhou^1^*, Yingzi Zhang^1^*, Jinyu Bai^1^*

^1^Department of Orthopedics, The Second Affiliated Hospital of Soochow University, Suzhou, Jiangsu 215004, China

^2^Department of Plastic and Aesthetic Surgery, The Second Affiliated Hospital of Soochow University, Suzhou, Jiangsu 215004, China

^3^Institute of Functional Nano & Soft Materials (FUNSOM), Soochow University, Suzhou, Jiangsu 215123, China

*Corresponding authors: Jinyu Bai: baijy@suda.edu.cn, Yingzi Zhang: [zhangyz@suda.edu.cn](mailto:zhangyz@suda.edu.cn), Xiaozhong Zhou: [zhouxz@suda.edu.cn](mailto:zhouxz@suda.edu.cn), Chao Wang: cwang@suda.edu.cn.

^#^: These authors contributed equally to this work

Table 1 Antibodies employed in this study.

Figure S1 Partial least squares discriminant analysis (PLS-DA) and Orthogonal partial least squares discriminant analysis (OPLS-DA).

Figure S2 Differentially expressed metabolites in tryptophan pathway identified by untargeted metabolomics.

Figure S3 Quantitative analysis of tryptophan metabolites.

Figure S4 Quantitative analysis of Figure 1I.

Figure S5 Influence of IPA on Bone Remodeling.

Figure S6 Biological safety of IPA in vivo.

Figure S7 In vitro cytotoxicity assessment evaluated by CCK8 assay.

Figure S8 Effect of various concentrations of IPA on osteoblast differentiation and osteoclast differentiation in vitro.

Figure S9 Quantitative analysis of qPCR and western blot of Figure 3.

Figure S10 Volcano plot.

Figure S11 Quantitative analysis of western blot of Figure 4.

Figure S12 Quantitative analysis of western blot of Figure 5.

Figure S13 Inhibition of AhR decreased the protein levels of β-catenin and IκBα.

Figure S14 Particle size distribution and zeta potential.

Figure S15 Engineered *Clostridium sporogenes* modulates the gut microbiota

Figure S16 Influence of Engineered *Clostridium sporogenes* (IPA CS) on Bone Remodeling.

Figure S17 Biological safety of CS and IPA CS in vivo.

Figure S18 Original data of western blots in the paper.

Figure S19 Original data of western blots in the paper.

Figure S20 Original data of western blots in the paper.

Table 2 Population information.

Table 3 Specific primer sequences for qPCR analysis.

**Table 1 Antibodies employed in this study.**

| **Antibodies** | **Manufacturer** | **Catalog Number** | **Dilutions** |
| --- | --- | --- | --- |
| Anti-Cathepsin K | Abcam | ab207086 | 1:1000 |
| Anti-SP7 | Abcam | ab209484 | 1:1000 |
| Anti-RUNX2 | Cell Signaling  Technology | #12556 | 1:1000 |
| Anti-β-catenin | Cell Signaling  Technology | #8480 | 1:1000 |
| Anti-β-TRCP | Cell Signaling  Technology | #4394 | 1:1000 |
| Anti-NFATc1 | Santa Cruz Biotechnology | sc-7294 | 1:200 |
| Anti-Cathepsin K | Servicebio | GB111276 | 1:200 |
| Anti-SP7 | Servicebio | GB111900 | 1:200 |
| Anti-ZO1 | Servicebio | GB12195 | 1:200 |
| Anti-Occludin | Servicebio | GB111401 | 1:500 |
| Anti-IκBα | Affinity | AF5002 | 1:1000 |
| Anti-AhR | Protintech | 28727-1-AP | 1:1000 |
| Anti-GAPDH | Protintech | 60004-1-Ig | 1:5000 |
| HRP-conjugated Goat Anti-Rabbit IgG(H+L) | Protintech | SA00001-2 | 1:5000 |
| HRP-conjugated Goat Anti-Mouse IgG(H+L) | Protintech | SA00001-1 | 1:5000 |
| The universal secondary antibody | Abmart | M21008 | 1:5000 |
| Rabbit IgG | Beyotime | A7016 | / |
| Alexa Fluor 488-Labeled Goat Anti-Rabbit IgG | Servicebio | GB25303 | 1:400 |
| CY3-Labeled Goat Anti-Rabbit IgG | Servicebio | GB21303 | 1:300 |


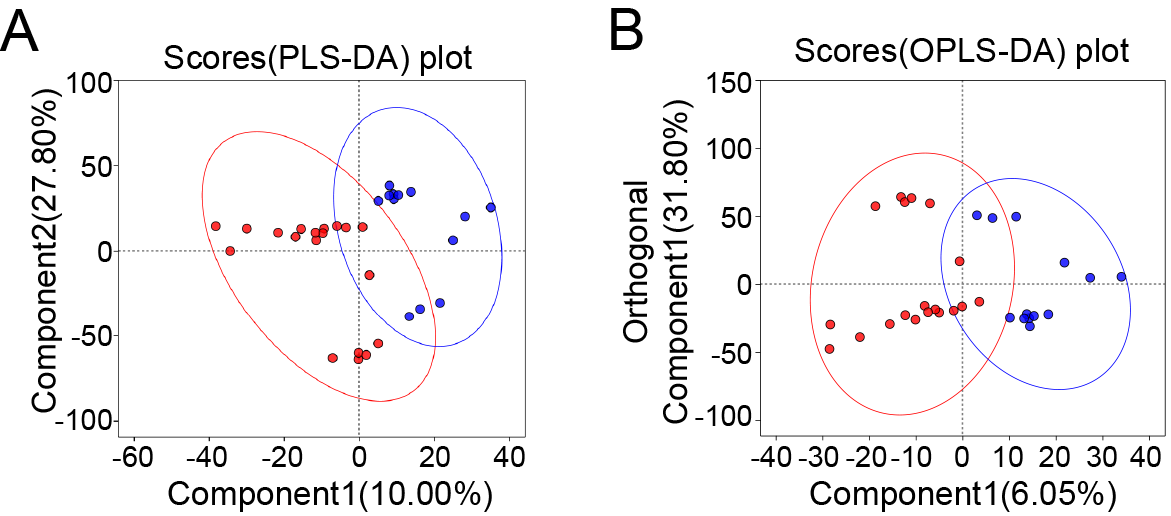


**Figure S1. Partial least squares discriminant analysis (PLS-DA) and Orthogonal partial least squares discriminant analysis (OPLS-DA)**

(A and B) Partial least squares discriminant analysis (PLS-DA), and orthogonal partial least squares discriminant analysis (OPLS-DA) show metabolic differences between 19 PMOP patients (PMOP, red) and 13 postmenopausal women with normal bone mass (Normal, blue).


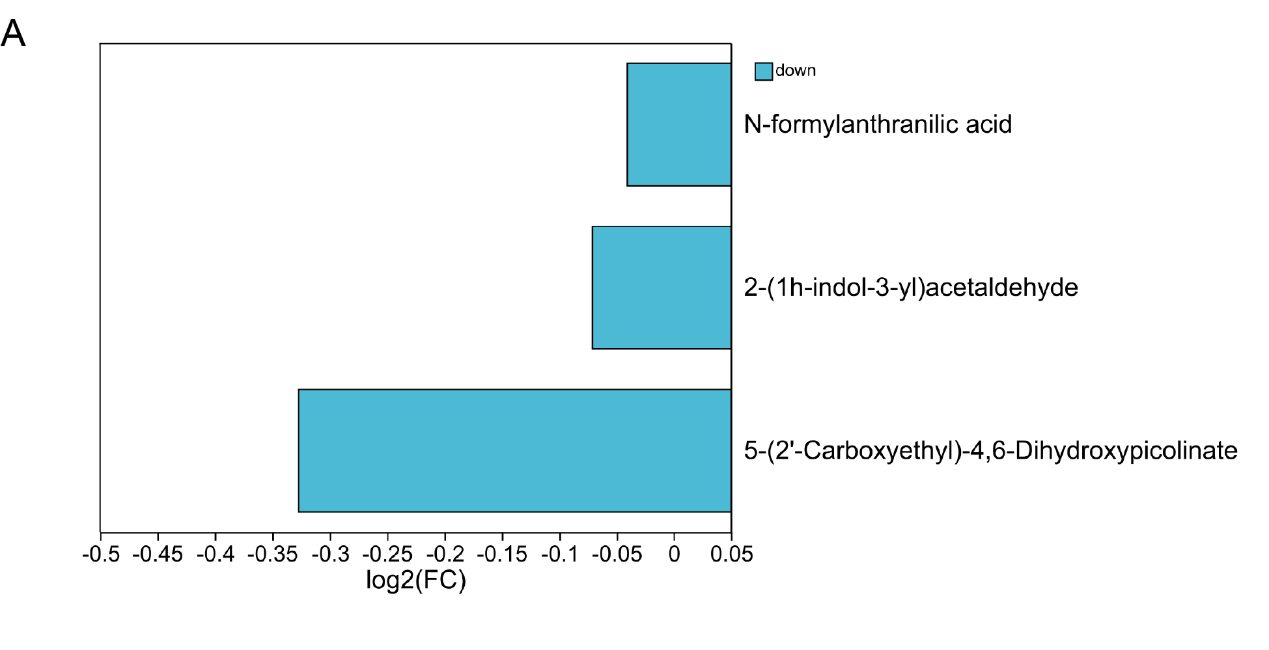


**Figure S2. Differentially expressed metabolites in tryptophan pathway identified by untargeted metabolomics**

(A) Untargeted metabolomics revealed significant downregulation of N-formylanthranilic acid, 2-(1H-indol-3-yl)acetaldehyde, and 5-(2'-carboxyethyl)-4,6-dihydroxypicolinate in tryptophan metabolism.


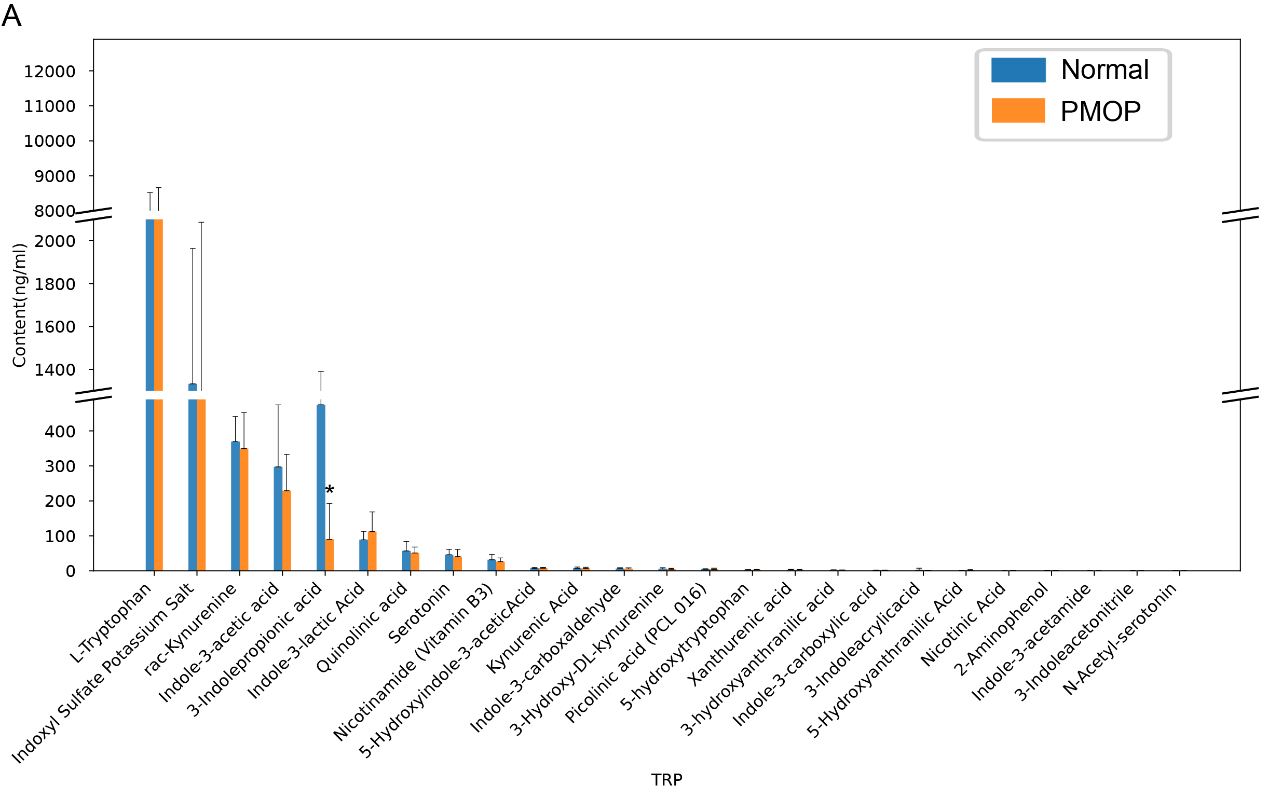


**Figure S3. Quantitative analysis of tryptophan metabolites**

(A) Serum tryptophan-targeted metabolomics analysis showing quantitative analysis of tryptophan metabolites between PMOP patients and the Normal group.

Data are presented as mean ± SD. Statistical significance was obtained by Mann–Whitney U test. Significance: **p* < 0.05.


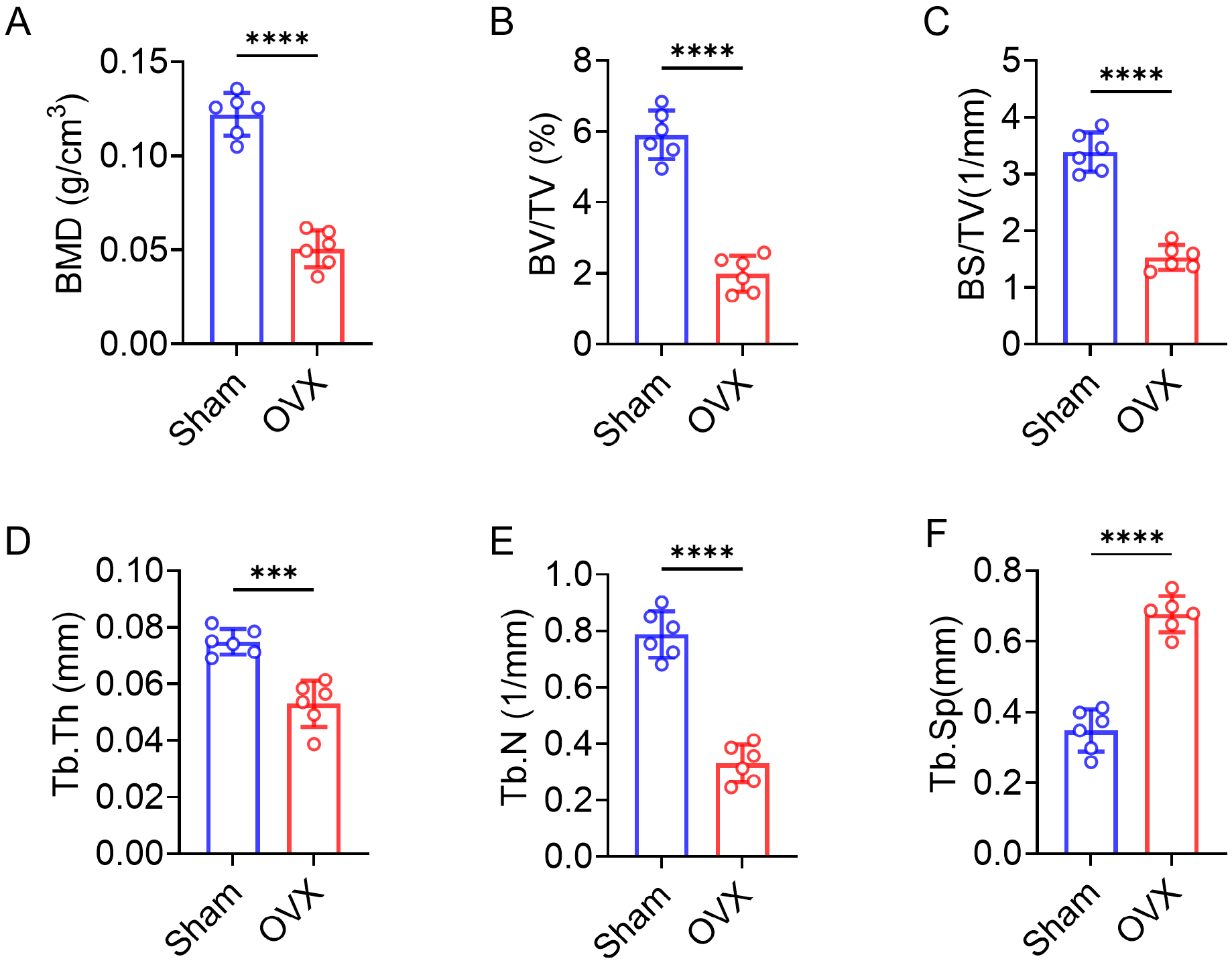


**Figure S4. Quantitative analysis** **of Figure 1I**

(A-F) Quantitative analysis of bone structural parameters in the distal femur (n=6).

Data are presented as mean ± SD. Statistical significance was obtained by Student t test (two-tailed). Significance: *****p* < 0.0001.


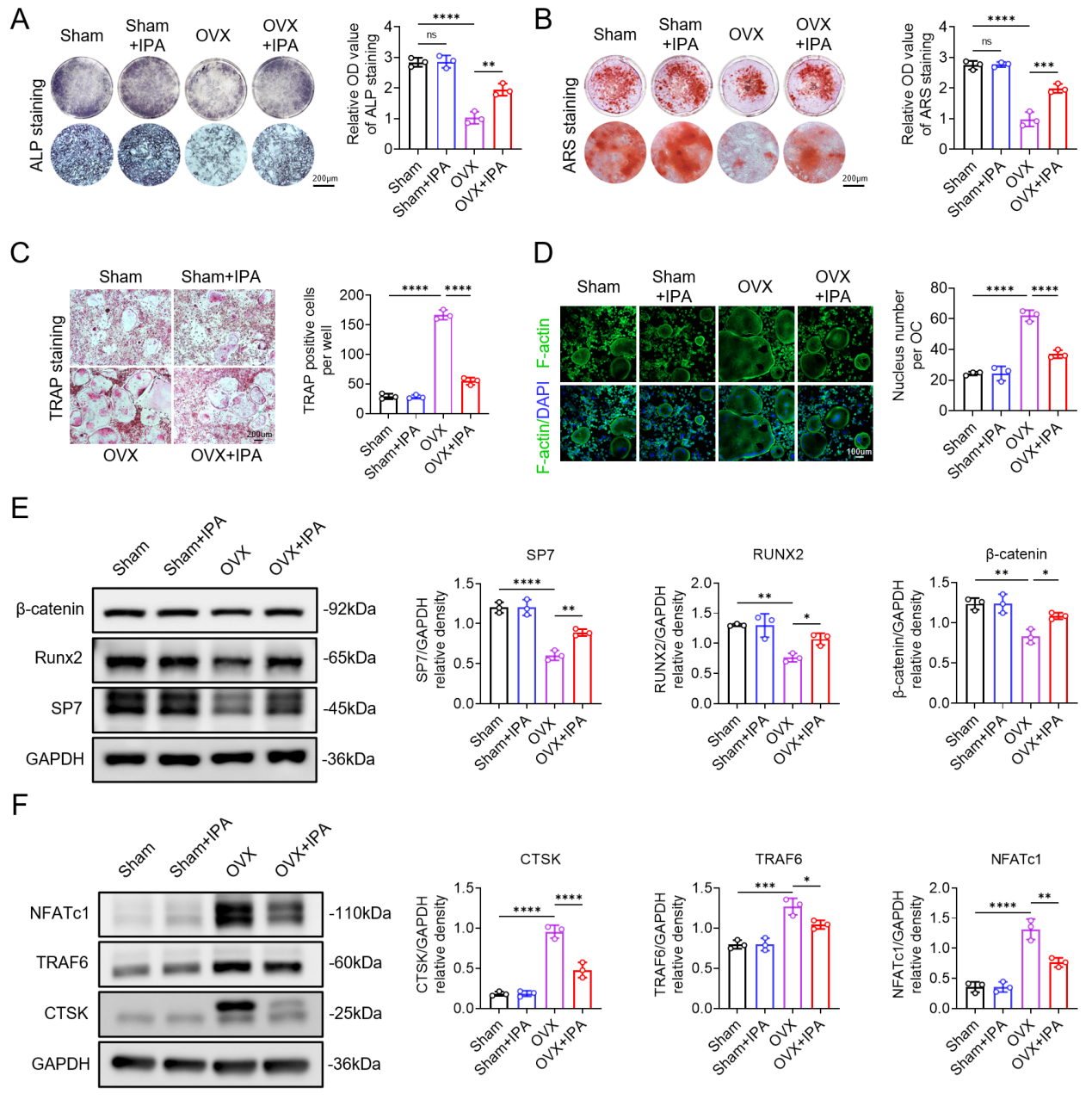


**Figure S5. Influence of IPA on Bone Remodeling**

(A) ALP staining of primary BMSCs from each group and quantitative analysis (Scale bar=200 µm. n=3).

(B) ARS staining of primary BMSCs from each group and quantitative analysis (Scale bar=200 µm. n=3).

(C) TRAP staining of primary monocytes from each group and quantitative analysis (Scale bar=200 µm. n=3).

(D) F-actin staining of primary monocytes from each group and quantitative analysis (Scale bar=100 µm. n=3).

(E) Representative western blot images and quantitative analysis of β-catenin, RUNX2, and SP7 protein expression during osteogenic differentiation of primary BMSCs from each group (n=3).

(F) Representative western blot images and quantitative analysis of NFATc1, TRAF6, and CTSK protein expression during osteoclast differentiation of primary monocytes from each group (n=3).

Data are presented as mean ± SD. Statistical significance was obtained by one-way ANOVA using the Tukey post-test. Significance: **p* < 0.05; ***p* < 0.01; ****p* < 0.001; *****p* < 0.0001; NS, non-significance.


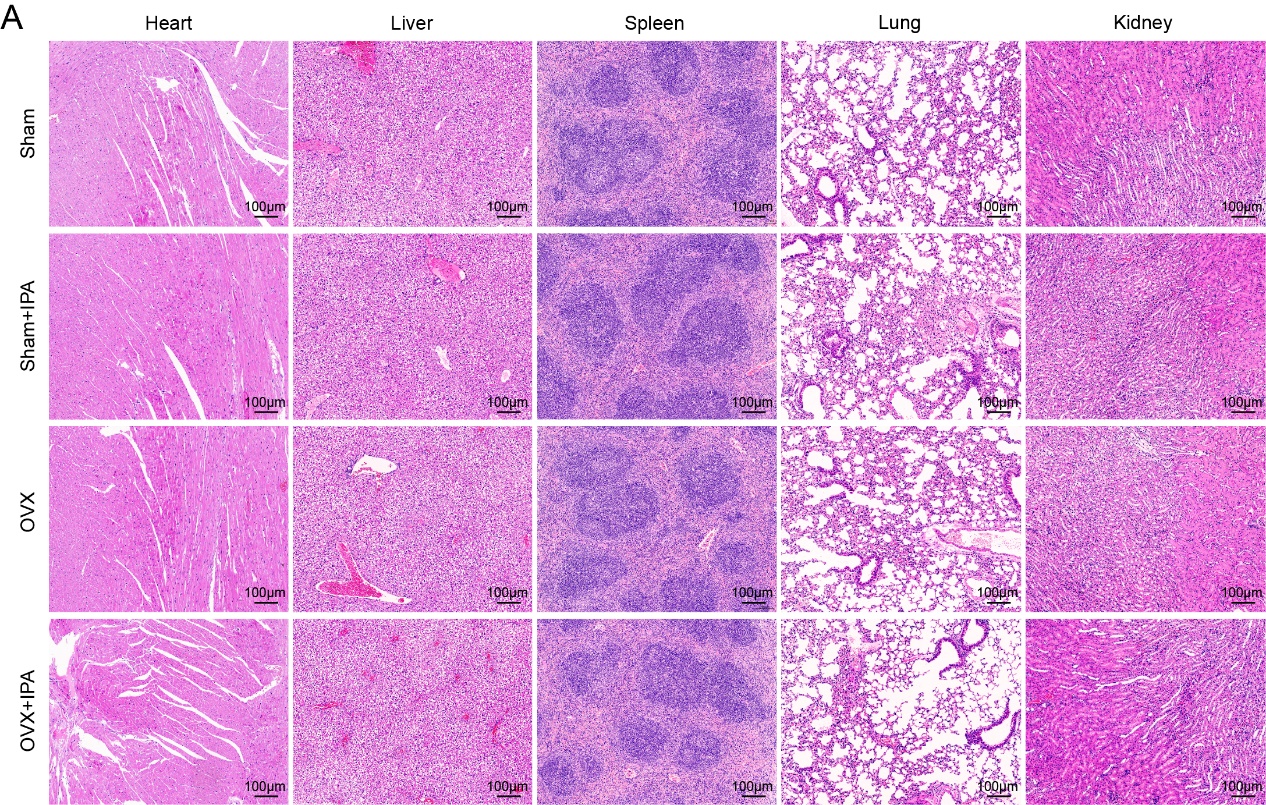


**Figure S6. Biological safety of IPA in vivo**

(A) H&E staining of major organs (heart, liver, spleen, lung, and kidney) in mice treated with IPA or vehicle control (Scale bar=100 µm. n=6).


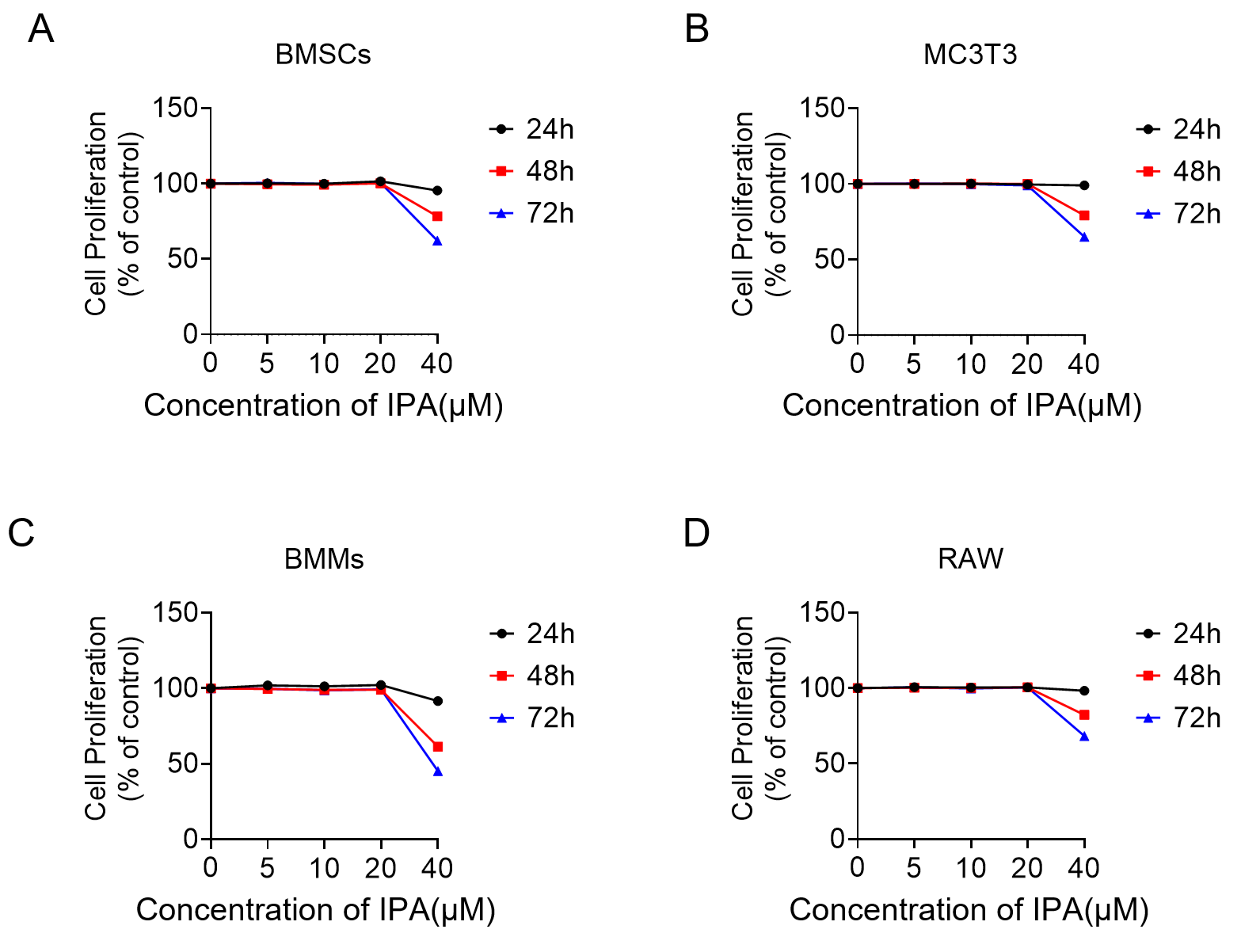


**Figure S7. In vitro cytotoxicity assessment evaluated by CCK8 assay**

(A and B) CCK8 assay showing the viability of osteoblast precursor cells treated with IPA for 24, 48 and 72 hours (n=3).

(C and D) CCK8 assay showing the viability of osteoclast precursor cells treated with IPA for 24, 48 and 72 hours. (n=3).

Data are means ± SD.


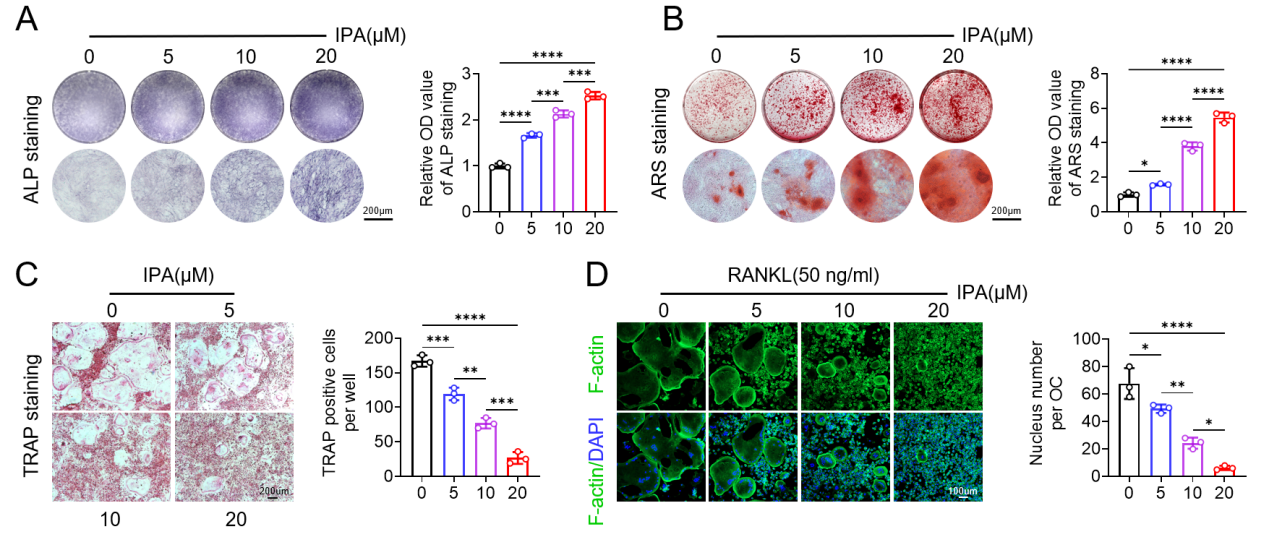


**Figure S8.** **Effect of various concentrations of IPA** **on osteoblast differentiation and osteoclast differentiation in vitro**

(A) ALP staining of BMSCs treated with various concentrations of IPA and quantitative analysis (Scale bar=200 µm. n=3).

(B) ARS staining of BMSCs treated with various concentrations of IPA and quantitative analysis (Scale bar=200 µm. n=3).

(C) TRAP staining of primary monocytes treated with varying concentrations of IPA and quantitative analysis (Scale bar=200 µm. n=3).

(D) F-actin staining of primary monocytes treated with different concentrations of IPA and quantitative analysis (Scale bar=200 µm. n=3).

Data are presented as mean ± SD. Statistical significance was obtained by one-way ANOVA using the Tukey post-test. Significance: **p* < 0.05; ***p* < 0.01; ****p* < 0.001; *****p* < 0.0001.


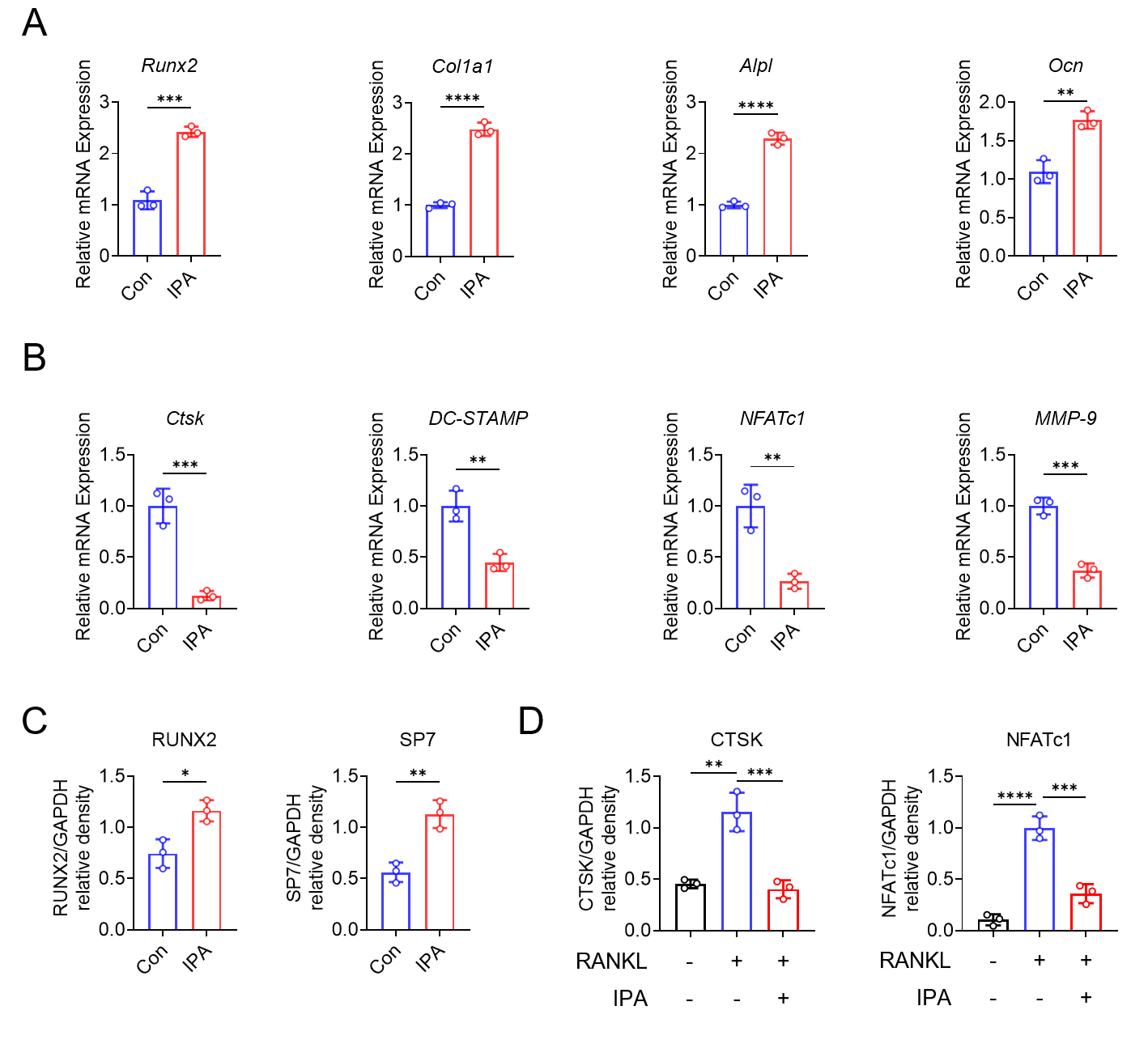


**Figure S9.** **Quantitative analysis of qPCR and western blot of Figure 3**

(A) qPCR analysis of mRNA expression levels of osteoblast-specific genes: *Runx2*, *Col1a1*, *Alpl*, and *Ocn* before and after IPA (20 μM) treatment (n=3).

(B) qPCR analysis of mRNA expression levels of osteoclast-specific genes: *Ctsk*, *DC-STAMP*, *NFATc1* and *MMP-9* before and after treatment with IPA (20 μM) (n=3).

(C) Quantitative analysis of RUNX2 and SP7 protein levels (n=3).

(D) Quantitative analysis of CTSK and NFATc1 protein levels (n=3).

Statistical significance was calculated by Student t test (two-tailed) (A-C) and one-way ANOVA (D). Significance: **p* < 0.05; ***p* < 0.01; ****p* < 0.001; *****p* < 0.0001.


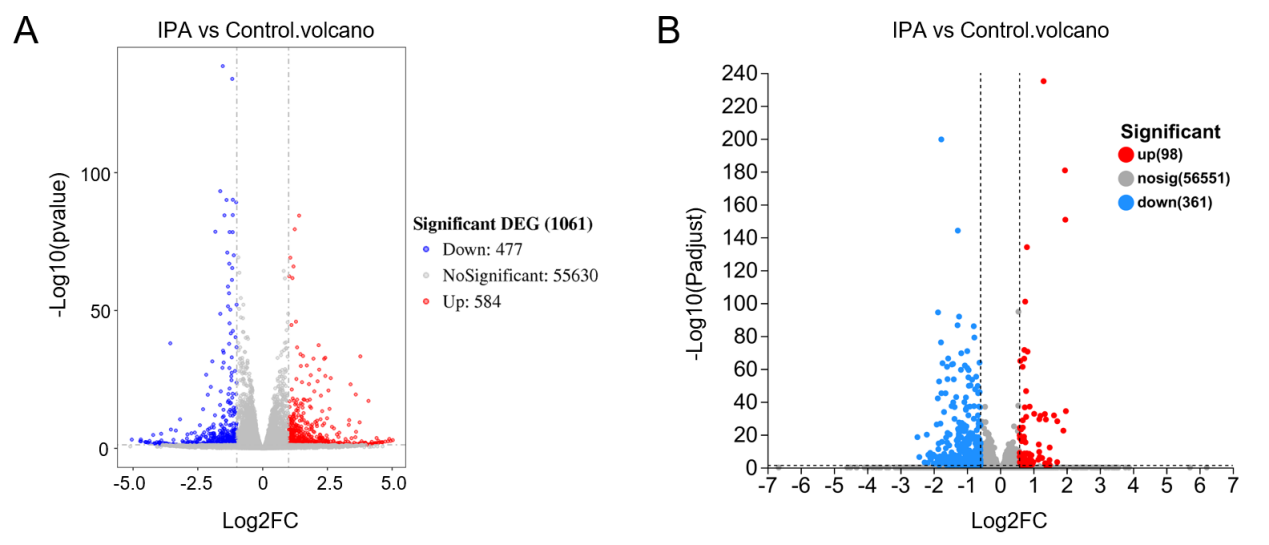


**Figure S10. Volcano plot**

(A)Volcano plot showing differential gene expression between control and IPA-treated groups in osteoblast differentiation, with a fold change threshold of 2 and a p-value of 0.05, identifying 584 upregulated and 477 downregulated genes.

(B)Volcano plot showing differential gene expression between control and IPA-treated groups in osteoclast differentiation, with a fold change threshold of 1.5 and a p-value of 0.05, identifying 98 upregulated and 361 downregulated genes.


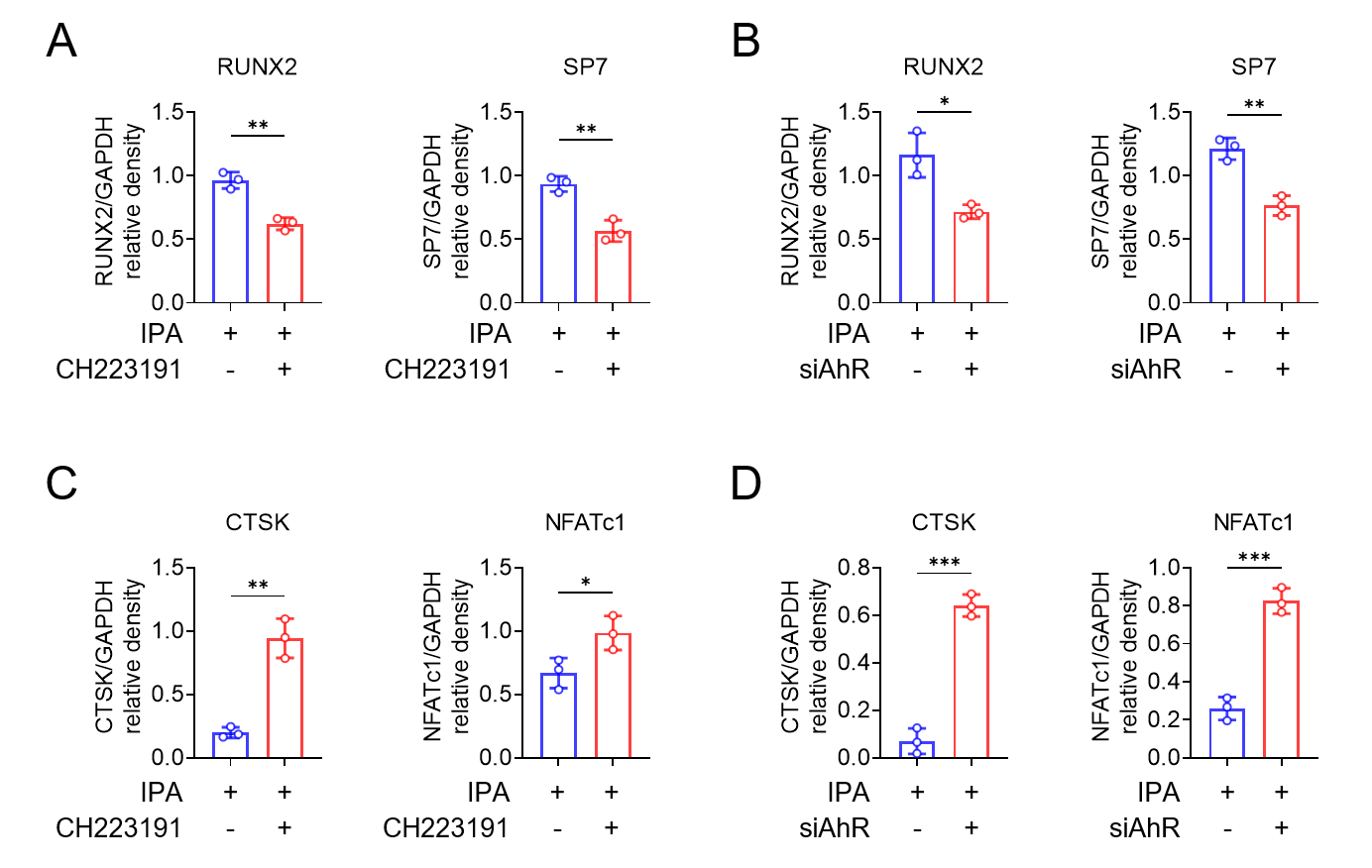


**Figure S11. Quantitative analysis of western blot of Figure 4**

(A) Quantitative analysis of RUNX2 and SP7 protein levels with or without CH223191 (n=3).

(B) Quantitative analysis of RUNX2 and SP7 protein levels with or without siAhR (n=3).

(C) Quantitative analysis of CTSK and NFATc1 protein levels with or without CH223191 (n=3).

(D) Quantitative analysis of CTSK and NFATc1 protein levels with or without siAhR (n=3).

Data are presented as mean ± SD. Statistical significance was obtained by Student t test (two-tailed). Significance: **p* < 0.05; ***p* < 0.01; ****p* < 0.001.


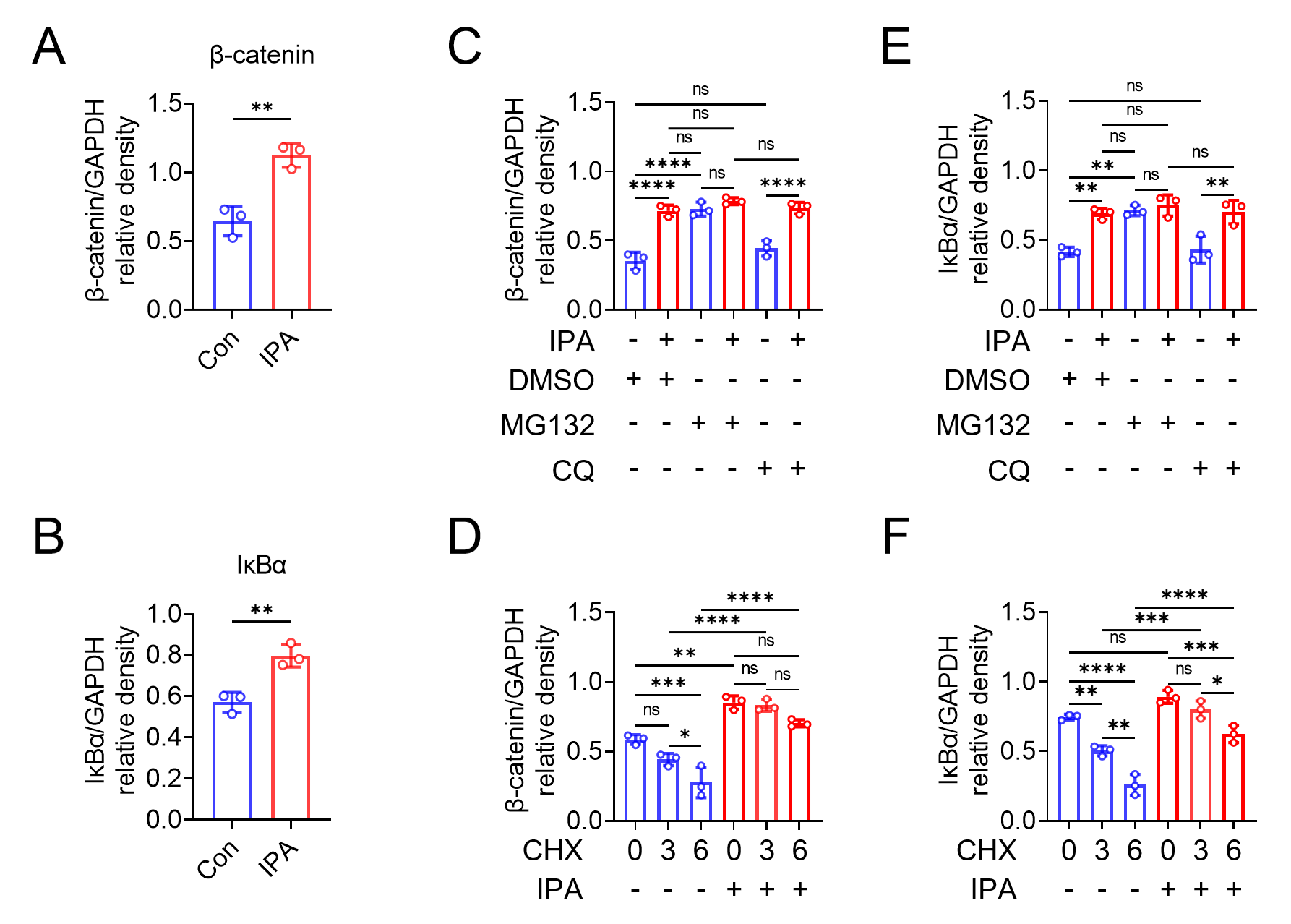


**Figure S12. Quantitative analysis of western blot of Figure 5**

(A) Relative protein quantification analysis of Figure 5C (n=3).

(B) Relative protein expression quantification of Figure 5D (n=3).

(C) Relative protein expression quantification of Figure 5E (n=3).

(D) Relative protein expression quantification of Figure 5F (n=3).

(E) Relative protein expression quantification of Figure 5G (n=3).

(F) Relative protein expression quantification of Figure 5H (n=3).

Statistical significance was calculated by Student t test (two-tailed) (A and B) and one-way ANOVA (C-F). Significance: **p* < 0.05; ***p* < 0.01; ****p* < 0.001; *****p* < 0.0001; NS, non-significance.


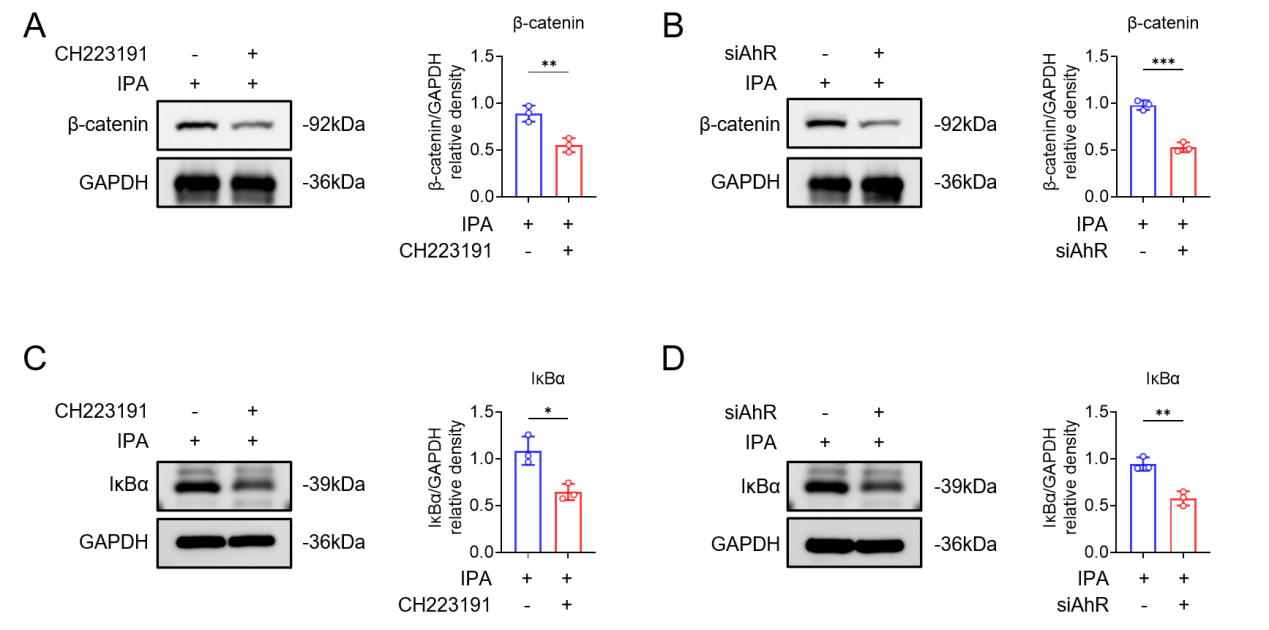
**Figure S13. Inhibition of AhR decreased the protein levels of β-catenin and IκBα**

(A) Representative western blot images and quantitative analysis of β-catenin protein level during osteogenic differentiation with or without CH223191 (n=3). (B) Representative western blot images and quantitative analysis of β-catenin protein level during osteogenic differentiation with or without siAhR (n=3).

(C) Representative western blot images and quantitative analysis of IκBα protein level during osteoclast differentiation with or without CH223191 (n=3). (D) Representative western blot images and quantitative analysis of IκBα protein level during osteoclast differentiation with or without siAhR (n=3).

Data are presented as mean ± SD. Statistical significance was obtained by Student t test (two-tailed). Significance: **p* < 0.05; ***p* < 0.01; ****p* < 0.001.

**
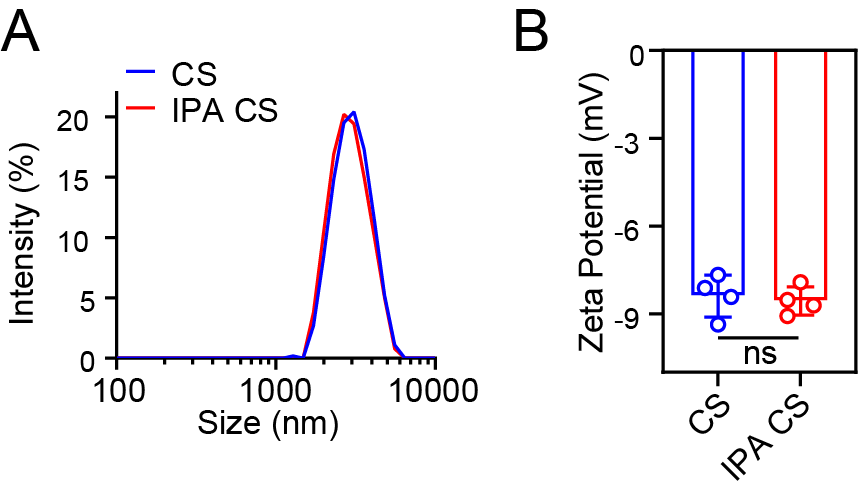
**

**Figure S14. Particle size distribution and zeta potential**

(A and B) Particle size distribution and zeta potential of CS and IPA CS, detected using dynamic light scattering (DLS) (n=4).

Data are presented as mean ± SD. Statistical significance was obtained by Student t test (two-tailed). Significance: NS, non-significance.


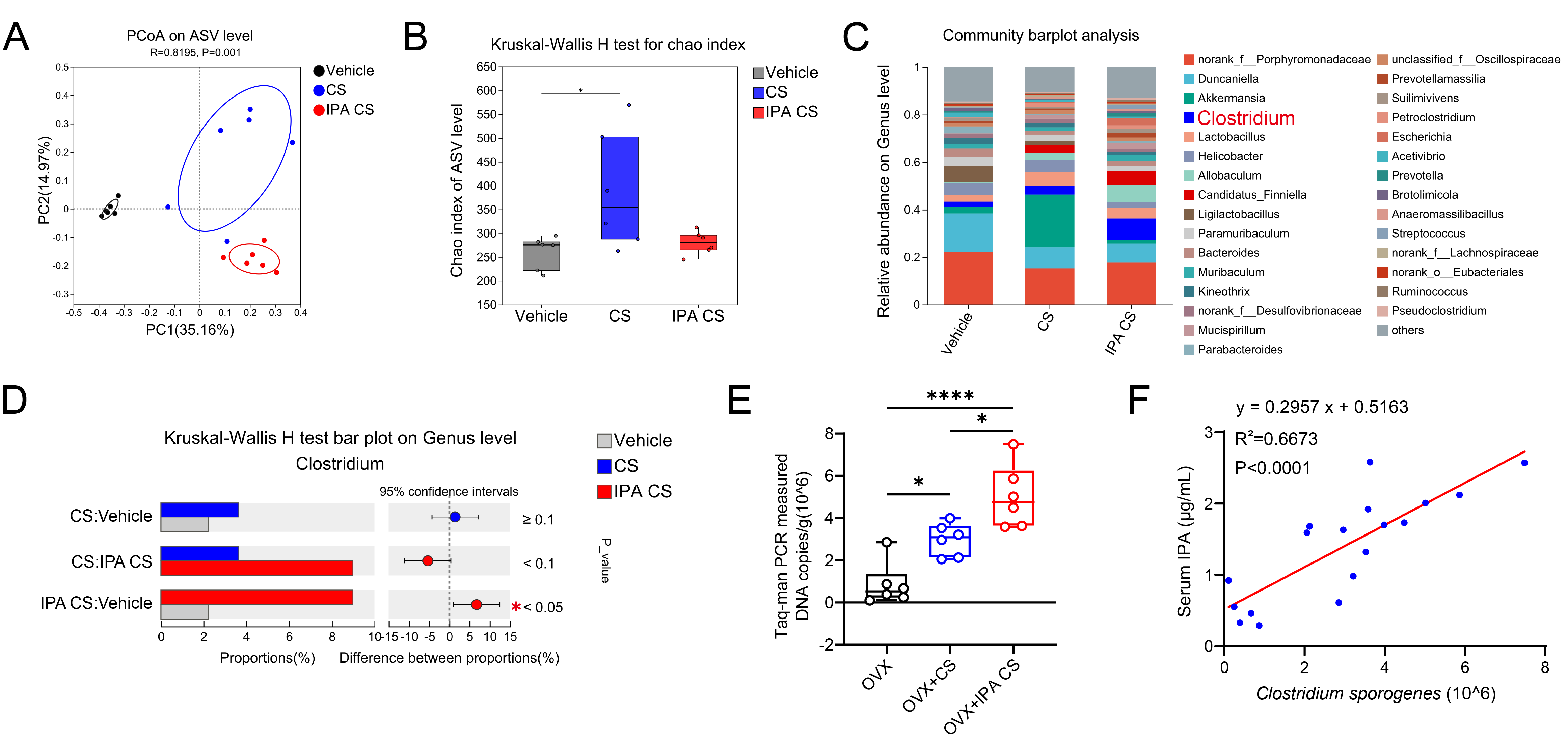


**Figure S15. Engineered *Clostridium sporogenes* modulates the gut microbiota**

(A) The principal coordinate analysis (PCoA) study showed variations of gut microbiota composition among three groups, with the statistical significance assessed by ANOSIM test. Each point represents an individual sample.

(B) Assessment of alpha-diversity using the Chao index among three groups.

(C) Comparison of gut microbiota composition at the genus level among each group.

(D) Kruskal-Wallis H test identified *Clostridium* in stool samples at the Genus level.

(E) Quantification of *Clostridium sporogenes* DNA in fecal samples by TaqMan quantitative PCR (qPCR).

(F) A scatter plot depicting the relationship between *Clostridium sporogenes* abundance and serum IPA concentration. Each point represents an individual subject. A trend of positive association is visible.

Data are presented as mean ± SD. Statistical significance was calculated by one-way ANOVA or Kruskal-Wallis H test. Significance: **p* < 0.05; *****p* < 0.0001. CS: *Clostridium sporogenes*. IPA CS: Engineered *Clostridium sporogenes*.


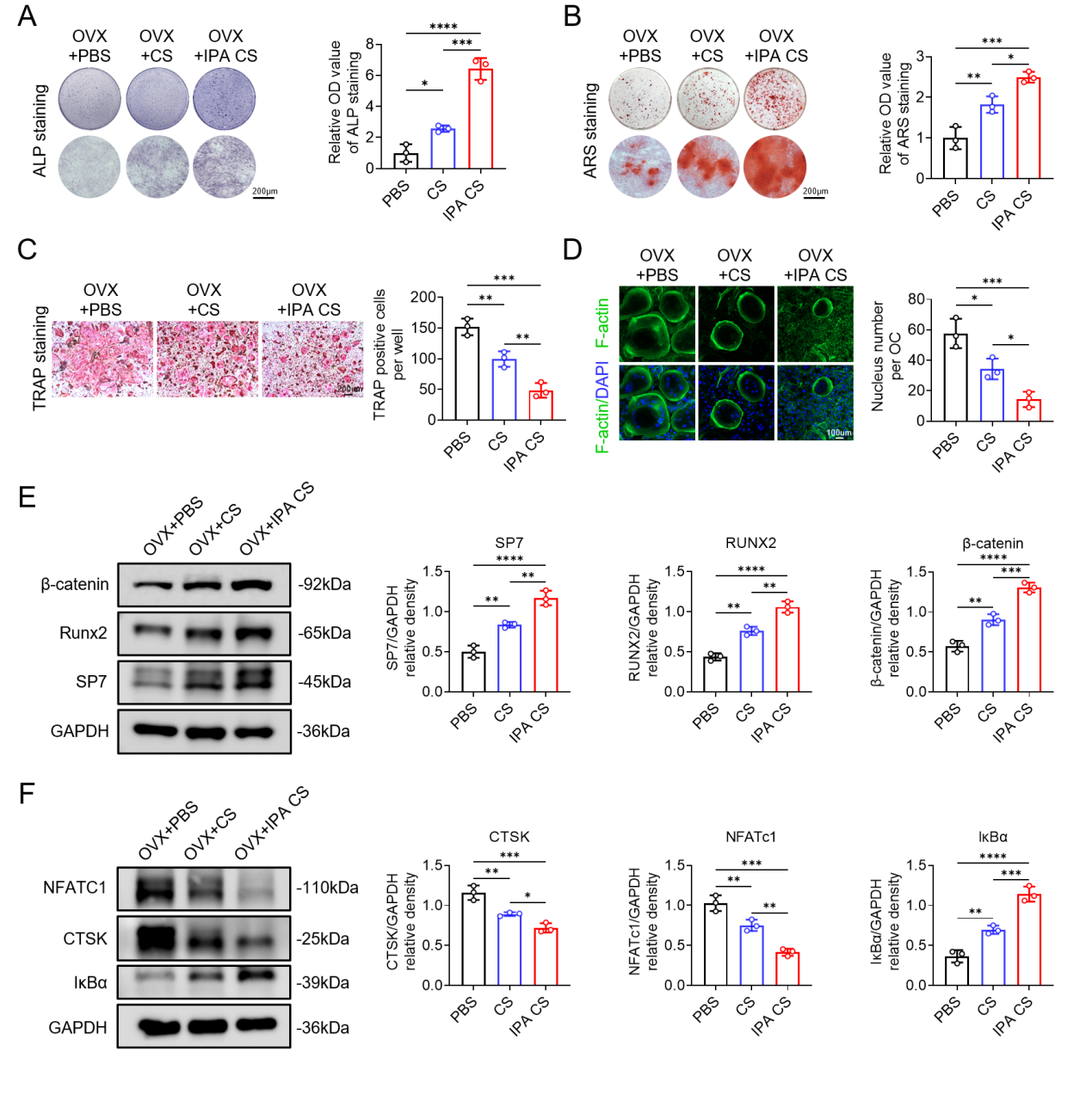


**Figure S16. Influence of** **Engineered** ***Clostridium sporogenes* (IPA CS) on Bone Remodeling**

(A) ALP staining of primary BMSCs from each group and quantitative analysis (Scale bar=200 µm. n=3).

(B) ARS staining of primary BMSCs from each group and quantitative analysis (Scale bar=200 µm. n=3).

(C) TRAP staining of primary monocytes from each group and quantitative analysis (Scale bar=200 µm. n=3).

(D) F-actin staining of primary monocytes from each group and quantitative analysis (Scale bar=100 µm. n=3).

(E) Representative western blot images and quantitative analysis of β-catenin, RUNX2, and SP7 protein expression during osteogenic differentiation of primary BMSCs from each group (n=3).

(F) Representative western blot images and quantitative analysis of NFATc1, CTSK, and IκBα protein expression during osteoclast differentiation of primary monocytes from each group (n=3).

Data are presented as mean ± SD. Statistical significance was calculated by one-way ANOVA. Significance: **p* < 0.05; ***p* < 0.01; ****p* < 0.001; *****p* < 0.0001. CS: *Clostridium sporogenes*. IPA CS: Engineered *Clostridium sporogenes*.


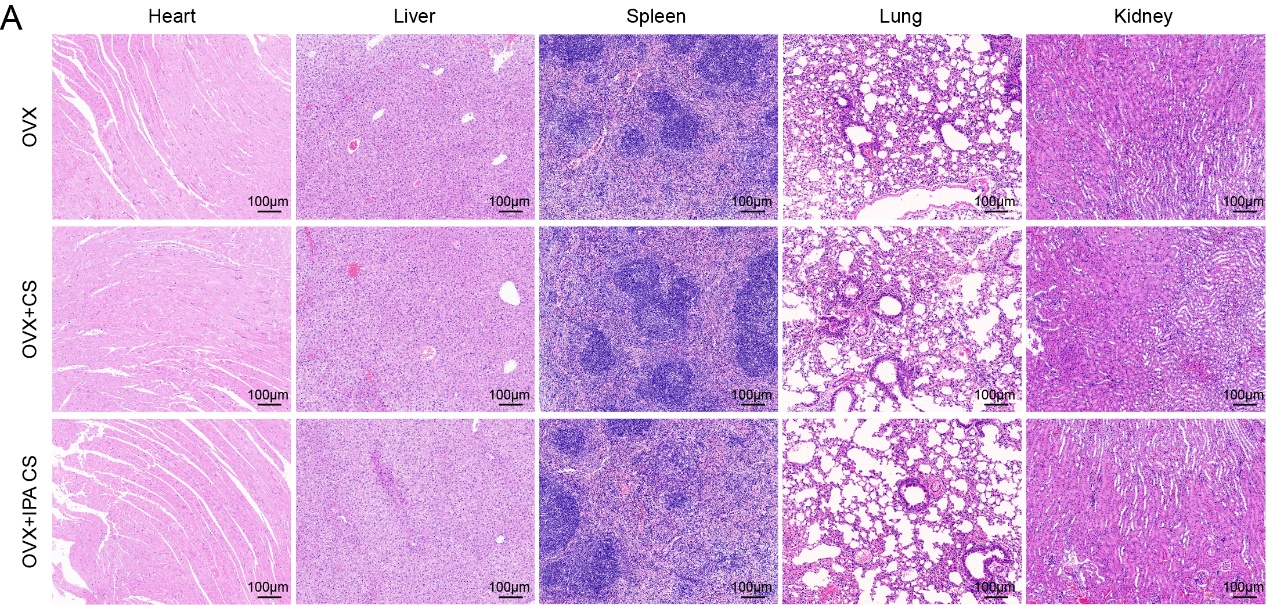


**Figure S17. Biological safety of CS and IPA CS in vivo**

(A) H&E staining of major organs (heart, liver, spleen, lung, and kidney) in mice treated with CS, IPA CS or vehicle control (Scale bar=100 µm. n=6).


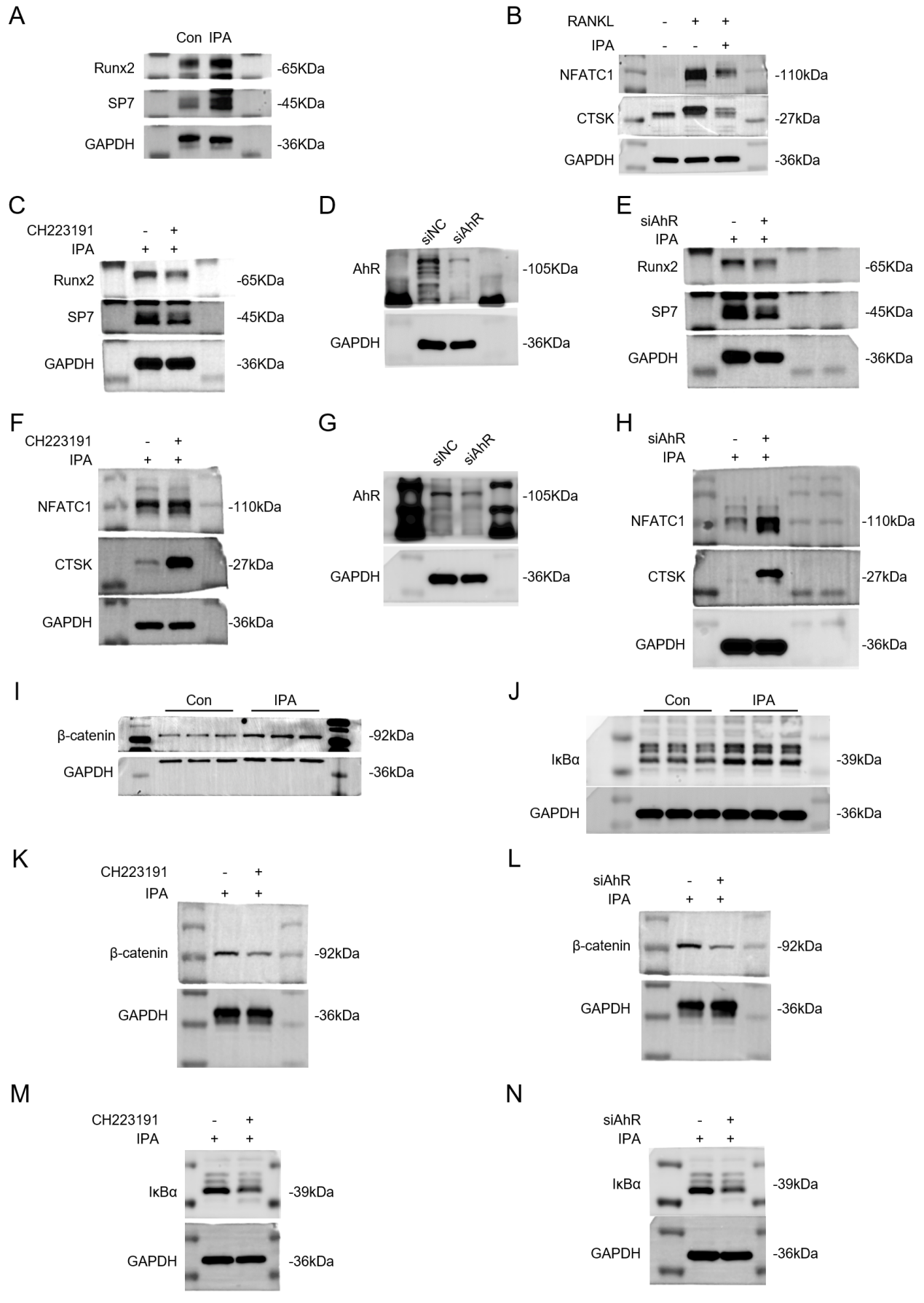


**Figure S18. Original data of western blots in the paper**

(A) Original western blot image for Figure 3H.

(B) Original western blot image for Figure 3I.

(C) Original western blot image for Figure 4C.

(D) Original western blot image for Figure 4D.

(E) Original western blot image for Figure 4G.

(F) Original western blot image for Figure 4J.

(G) Original western blot image for Figure 4K.

(H) Original western blot image for Figure 4N.

(I) Original western blot image for Figure 5C.

(J) Original western blot image for Figure 5D.

(K) Original western blot image for Figure S11A.

(L) Original western blot image for Figure S11B.

(M) Original western blot image for Figure S11C.

(N) Original western blot image for Figure S11D.


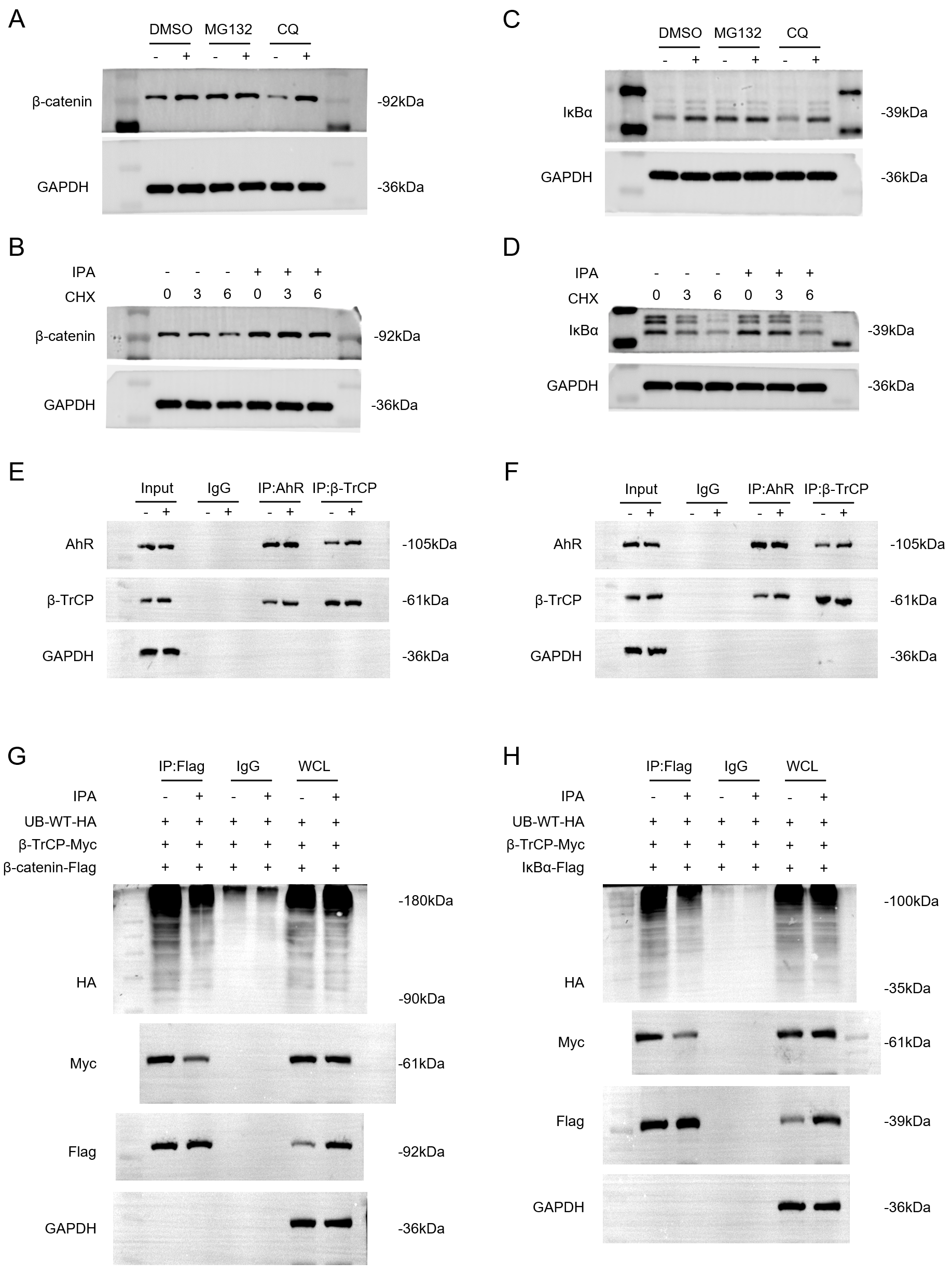


**Figure S19. Original data of western blots in the paper**

(A) Original western blot image for Figure 5E.

(B) Original western blot image for Figure 5F.

(C) Original western blot image for Figure 5G.

(D) Original western blot image for Figure 5H.

(E) Original western blot image for Figure 5J.

(F) Original western blot image for Figure 5K.

(G) Original western blot image for Figure 5L.

(H) Original western blot image for Figure 5M.


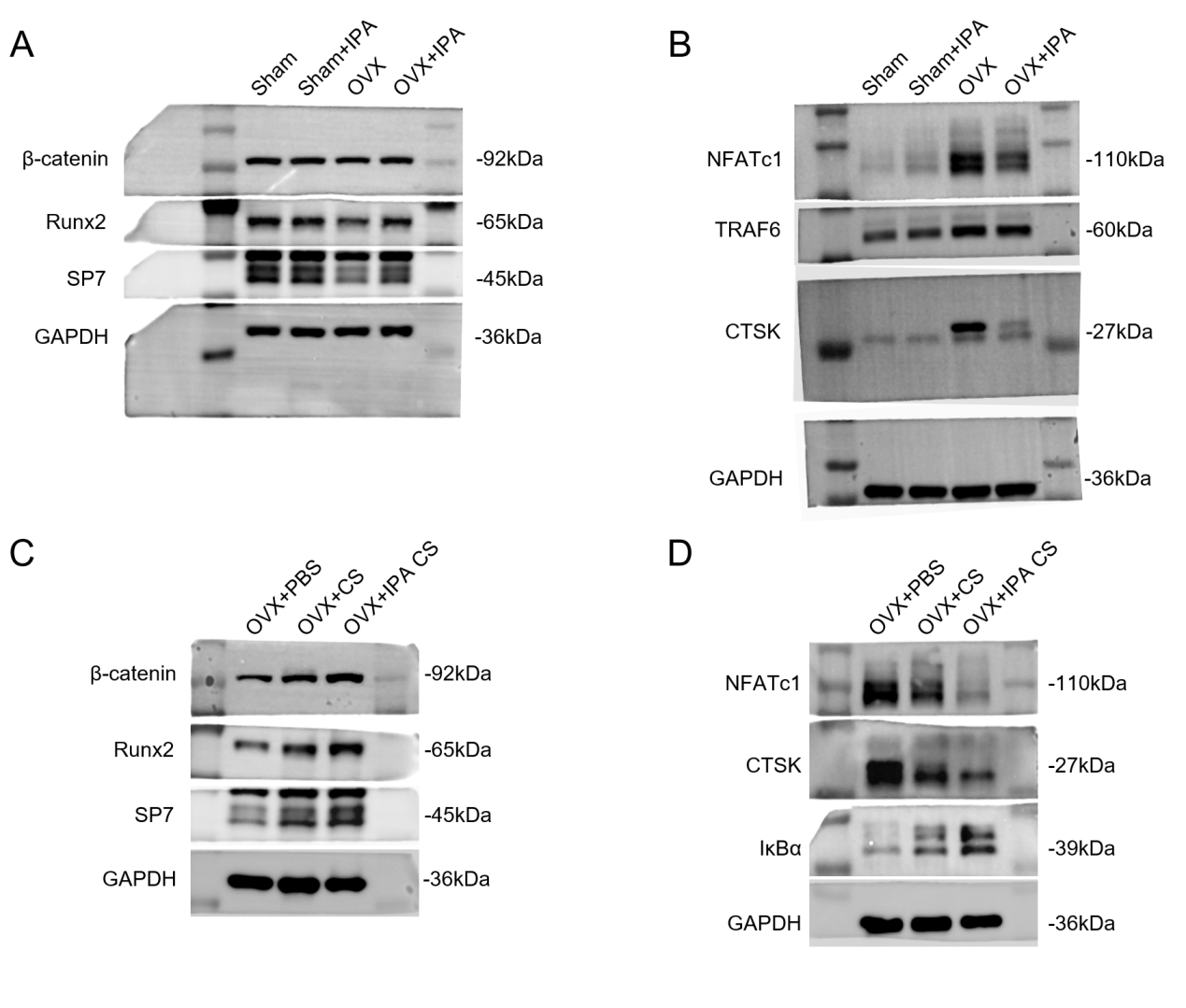
**Figure S20. Original data of western blots in the paper**

(A) Original western blot image for Figure S4E.

(B) Original western blot image for Figure S4F.

(C) Original western blot image for Figure S13E.

(D) Original western blot image for Figure S13F.

**Table 2 Population information.**

| Parameter | Normal (n=13) | PMOP (n=19) | *p* value |
| --- | --- | --- | --- |
| Age (years) | 63.23±5.069 | 65.32±4.911 | 0.2534 |
| Height (cm) | 157.4±6.899 | 154.8±4.658 | 0.2218 |
| Weight (kg) | 59.54±5.695 | 56.58±6.552 | 0.1964 |
| BMI (kg/m²) | 24.16±3.105 | 23.62±2.719 | 0.6057 |
| BMD (T value) | -0.1769±0.9639 | -3.468±0.9226 | **<0.001** |
| Fasting glucose (mmol/L) | 5.329±0.4177 | 5.120±0.6049 | 0.2884 |
| Total cholesterol (mmol/L) | 4.431±0.8471 | 4.538±0.9019 | 0.7377 |
| PTH (pg/mL) | 50.81±14.79 | 51.61±17.64 | 0.8940 |
| Creatinine clearance (ml/min) | 98.85±11.39 | 93.68±10.22 | 0.1903 |
| 25-hydroxyvitamin D (nmol/L) | 18.39±9.191 | 14.52±7.088 | 0.1885 |
| β-CTX (pg/mL) | 595.5±221.6 | 843.4±320.8 | **0.0221** |

Notes: “Normal” represents postmenopausal women with normal bone mass and “PMOP” represents postmenopausal women with osteoporosis. Data are presented as mean ± SD. Statistical significance was obtained by Student t test (two-tailed). **p* < 0.05, ****p* < 0.001. PTH: Parathyroid Hormone.

**Table 3 Specific primer sequences for qPCR analysis.**

| **Gene** | **Primer sequences (5’-3’)** |
| --- | --- |
| *Ctsk* | Forward CAGCAGGATGTGGGTGTTCAA  Reverse GCTTCTGGTGAGTCTTCTTCC |
| *NFATc1* | Forward TATATGAGCCCATCCTTGCCT  Reverse GCTGCCTTCCGTCTCATAGTG |
| *MMP-9* | Forward TCCATCCTCCTCCCTCCACTT  Reverse GCGGTACAAGTATGCCTGGAA |
| *DC-STAMP* | Forward CAGGGAGAATGAGGCTCTGGA  Reverse CACCGAAAGGAAGGCCACAAA |
| *NFKBIA* | Forward GAGCTCCGAGACTTTCGAGG  Reverse AGACACGTGTGGCCATTGTA |
| *Runx2* | Forward GGACGAGGCAAGAGTTTCACC  Reverse TTAGAGTCATCAAGCTTCTGTCTG |
| *Col1a1* | Forward CCCTGGTCCCTCTGGAAATG  Reverse GGACCTTTGCCCCCTTCTTT |
| *Alpl* | Forward CGGACAATGAGATGCGCCC  Reverse AGACATAGTGGGAGTGCTTGTG |
| *Ocn* | Forward CTTGAAGACCGCCTACAAAC  Reverse GCTGCTGTGACATCCATAC |
| *CTNNB1* | Forward GTCAGTGCAGGAGGCCGA  Reverse CTCCATCAGGTCAGCTTGAGT |
